# Cultivation-independent and cultivation-dependent metagenomes reveal genetic and enzymatic potential of microbial community involved in the degradation of a complex microbial polymer

**DOI:** 10.1186/s40168-020-00836-7

**Published:** 2020-06-01

**Authors:** Ohana Y. A. Costa, Mattias de Hollander, Agata Pijl, Binbin Liu, Eiko E. Kuramae

**Affiliations:** 1 Netherlands Institute of Ecology (NIOO-KNAW), Department of Microbial Ecology, Droevendaalsesteeg 10, 6708 PB Wageningen, Netherlands; 2 Institute of Biology (IBL), Leiden University, Leiden, The Netherlands; 3 Center for Agricultural Resources Research, Institute of Genetics and Developmental Biology, Chinese Academy of Sciences, Shijiazhuang, 050021 Hebei China

## Abstract

**Background:**

Cultivation-independent methods, including metagenomics, are tools for the exploration and discovery of biotechnological compounds produced by microbes in natural environments. Glycoside hydrolases (GHs) enzymes are extremely desired and important in the industry of production for goods and biofuel and removal of problematic biofilms and exopolysaccharide (EPS). Biofilms and EPS are complex, requiring a wide range of enzymes for a complete degradation. The aim of this study was to identify potential GH microbial producers and GH genes with biotechnological potential, using EPS-complex structure (WH15EPS) of *Acidobacteria Granulicella* sp. strain WH15 as an enrichment factor, in cultivation-independent and cultivation-dependent methods. We performed stable isotope probing (SIP) combined with metagenomics on topsoil litter amended with WH15EPS and coupled solid culture-EPS amended medium with metagenomics.

**Results:**

SIP metagenome analysis of the soil litter demonstrated that phyla *Proteobacteria*, *Actinobacteria*, *Acidobacteria*, and *Planctomycetes* were the most abundant in WH15EPS amended and unamended treatments. The enrichment cultures in solid culture medium coupled to metagenomics demonstrated an enrichment in *Proteobacteria*, and the metagenome assembly of this enrichment cultures resulted in 4 metagenome-assembled genomes (MAGs) of microbes with low identity (42–86%) to known microorganisms. Among all carbohydrate-active enzymes (CAZymes) retrieved genes, glycoside transferase (GT) was the most abundant family, either in culture-independent or culture-based metagenome datasets. Within the glycoside hydrolases (GHs), GH13 was the most abundant family in both metagenome datasets. In the “heavy” fraction of the culture-independent metagenome SIP dataset, GH109 (α-N-acetylgalactosaminidases), GH117 (agarases), GH50 (agarases), GH32 (invertases and inulinases), GH17 (endoglucanases), and GH71 (mutanases) families were more abundant in comparison with the controls. Those GH families are affiliated to microorganism that are probably capable to degrade WH15EPS and potentially applicable for biofilm deconstruction. Subsequent in culture-based metagenome, the assembled 4 MAGs (unclassified *Proteobacteria*) also contained GH families of interest, involving mannosidases, lysozymes, galactosidases, and chitinases.

**Conclusions:**

We demonstrated that functional diversity induced by the presence of WH15EPS in both culture-independent and culture-dependent approaches was enriched in GHs, such as amylases and endoglucanases that could be applied in chemical, pharmaceutical, and food industrial sectors. Furthermore, WH15EPS may be used for the investigation and isolation of yet unknown taxa, such as unclassified *Proteobacteria* and *Planctomycetes*, increasing the number of current cultured bacterial representatives with potential biotechnological traits.

Video Abstract

## Background

Metagenomics approach allows the access to a microbial genetic pool that is not reachable through classical microbial cultivation techniques. Therefore, the cultivation-independent methods have long been used as a tool for the exploration and discovery of biotechnological compounds produced by microbes in natural environments, in particular the detection of potential enzymes and other products of economic significance [1]. Culture-independent approaches allowed the clarification of potential microbial roles; however, culture-based studies are still needed for the comprehension of microbial characteristics and phenotypes [2]. The use of metagenomics has boosted industrial production systems and enzyme bioprospecting [3], particularly in animal guts [4], although other types of ecosystems, such as forest litter, remain underexplored.

Glycoside hydrolases (GHs) are among the industrially important enzymes that are extensively searched through metagenomics, as they are extremely desired and important in food and other industrial sectors [4–7]. Those enzymes are employed for brewing, baking, production of syrups, food processing, texture, flavoring, as well as the production of dairy and fermented foods [8]. GHs are also necessary for the production of biofuels, by converting cellulose and lignocellulosic biomass into sugars that can be fermented by microorganisms into bioethanol [9].

An alternative application of GHs is the degradation of polysaccharides for the removal of biofilms. Exopolysaccharides are the main and most studied components of extracellular polymeric substances (EPS), biopolymers synthesized by a wide range of strains of microorganisms [10]. EPS are the constituents that preserve the tridimensional structure of biofilms, maintaining internal cohesion and promoting adhesion to surfaces [11]. The elimination of biofilms is important for human health in general, because those structures are implicated in several diseases, causing problems for instance in hospitals and in food processing industries [2]. Furthermore, enzymatic removal of biofilms is superior to the use of conventional cleaning agents, which are not eco-friendly, producing toxic residues, and erosion of equipment [2]. Enzymes are an environmentally friendly alternative due to their biodegradable nature [12]. EPS and biofilms are complex, requiring a wide range of enzymes for a complete degradation [11]; however, enzymes such as lysozyme, amylases, dispersin B, and alginate lyase are already used for biofilm removal or inhibition in food and pharmaceutical industries [2]. More than 50% of the current industrial enzymes are produced by microorganisms, such as strains of *Bacillus* and *Aspergillus*, while around 15% are derived from plants [12]. Furthermore, microbial enzymes with potential applications were obtained from habitats such as hydrothermal vents [13], arctic tundra [14], cow rumen [15], and termite guts [16].

The main goal of our study was to use a microbial EPS to target microbes and functions involved in EPS degradation in microcosm experiment with temperate forest litter and in culture medium. Plant litter is mostly composed of recalcitrant biopolymers, which are sources of carbon, energy, and nutrients for microbial communities living in litters layers [17]. Cellulose, hemicellulose, and pectin are the major components of plant cell walls. Cellulose is the most abundant plant cell wall component (40–50% of the dry weight), composed of β (1→4) linear chains of D-glucose residues. Hemicelluloses (20–30% of plant dry weight) are mostly composed of xylan, xyloglucan, β-glucan, and mannan as well as other oligosaccharides. Pectins (10–30% of plant dry weight) contain homogalacturonan, xylogalacturonan, and rhamnogalacturonan [18]. Due to their complexity, the breakdown of plant cell wall components requires a wide range of enzymes, produced by the microorganisms during litter decomposition process [19]. Therefore, it is an interesting environment for the retrieval of complex polysaccharide-degrading enzymes. On the other hand, the microbial community in forest ecosystems is dominated by *Acidobacteria* [20], which phylum members are linked to carbon degradation [21]. *Acidobacteria* isolates belonging to *Granulicella* sp. from forest litter are described to produce large amounts of EPS [19]. The genus *Granulicella* is not a human pathogen [22], and the unique composition of its EPS is interesting for the retrieval of a wide range of glycoside hydrolase genes that could be applied in the industry for several processes [23]. The EPS of the *Acidobacteria Granulicella* sp. strain WH15 (WH15EPS) has a more complex composition than most commercially available microbial polymers. It is composed of 7 monosaccharides (mannose, glucose, galactose, xylose, rhamnose, glucuronic, and galacturonic acids) [23], while other known EPS are composed of maximum 4 different monosaccharides [24]. The degradation of WH15EPS would require a broader range of enzymes than other EPS; therefore, the application of WH15EPS to topsoil-litter samples would promote the enrichment of a wider range of GHs. The use of EPS as a carbon source by active microorganisms can be investigated with stable isotope probing (SIP). SIP is a robust technique that evaluates the incorporation of compounds labeled with heavy isotopes, for instance ^13^C, ^18^O, and ^15^N, into the cell components of microorganisms metabolizing a specific substrate [25]. Hence, SIP identifies the active microorganisms involved in the metabolism of a specific labeled compound. It has been successfully applied for the study of microorganisms incorporating several compounds, such as methanol, phenol [26, 27], and others [28].

The aim of this study was to identify potential GH microbial producers and GH genes with biotechnological potential, using EPS of *Acidobacteria Granulicella* sp. strain WH15 (WH15EPS) as an enrichment factor, in cultivation-independent and cultivation-dependent methods. We performed stable isotope probing (SIP) combined with metagenomics on topsoil litter amended with WH15EPS and coupled solid culture-EPS amended medium with metagenomics.

## Results

### Overview of the metagenome data

#### SIP metagenome

After quality control filtering, a total of 18,762,958 reads were maintained for further analysis, with an average of 1,563,580 reads per sample. A total of 1,209,745 ORFs were predicted for functional annotation, and approximately 50% of these ORFs were classified using KEGG and COG databases. The sequencing statistics are in Table 1.

**Table 1:** SIP shotgun metagenomics sequencing statistics for each treatment. Average from 4 replicates

Statistics	Unamended Control	EPS amended	“Heavy” fraction
Number of reads	1,590,046	1,591,447	1,509,247
Number of contigs	82,370.25	78,474.5	92,521.75
Longest contig (bp)	2,651	3,645.25	9,414.75
N50	466	473	542
Mapping (%)	18.4	19.6	32.9
Number of ORFs	96,141	91,272	115,023.3

*Unamended Control* incubation without WH15EPS, *EPS amended* incubation containing ^12^C-WH15EPS, “Heavy” fraction, “heavy” fraction of incubations containing ^13^C-WH15EPS

#### Community composition SIP metagenome based on SSU rRNA and ORF classification

**Fig. 1:**
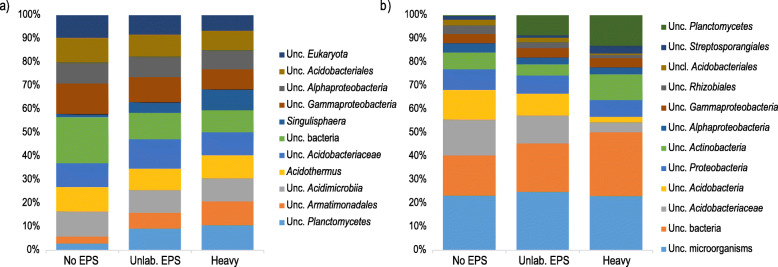
Taxonomic composition and relative abundance of microbial groups at genus level in SIP metagenome treatments based on **a** SSU rRNA gene taxonomic classification and **b** ORF taxonomic classification. Only the ten most abundant groups for each treatment are displayed. Average abundances of 4 replicates. Unc.: unclassified. No EPS: incubation without WH15EPS. Unlab.: EPS-incubation containing ^12^C-WH15EPS. Heavy: “heavy fraction” of incubations containing ^13^C-WH15EPS

Taxonomic annotation based on SSU rRNA annotation demonstrated that bacteria, fungi, and archaea accounted for approximately 84%, 4%, and 2% of the sequences, respectively. At phylum level, 17 bacterial groups, 5 fungal groups, and 3 archaeal groups were observed in all the samples. The most abundant groups at phylum level belonged to domain *Bacteria* (Additional file 1: Supplementary Figure S1a). *Proteobacteria* was the most abundant phylum in all treatments (26.4–28% of the sequences), followed by *Actinobacteria* (14.5–17.5% of the sequences). In both unamended and ^12^C-EPS-amended control treatments, *Acidobacteria* was the third most abundant group (14.5–15.8% of the sequences), while in the “heavy” fraction samples, *Planctomycetes* was the third most abundant phylum (16.45% of the sequences) (Additional file 1: Supplementary Figure S1a). At genus level, we observed 167 groups in all samples, of which 110 were unclassified groups. “Unclassified *Bacteria*” was the most abundant group in the unamended control (3.5% of the sequences), while “unclassified *Acidobacteriaceae*” (2.6% of the sequences) was the most abundant in the ^12^C-EPS-amended control (Fig. 1a). In labeled samples, the predominant group was “unclassified *Planctomycetes*” (3.2% of the sequences) (Fig. 1a). Among the 10 most abundant groups, only 2 classified genera were observed: *Acidothermus* (1.8–2.9% of the sequences) and *Singulisphaera* (0.2–2.6% of the sequences) (Fig. 1a). Similarly, the taxonomic composition of the ORF-based analysis was dominated by domain *Bacteria*, with an average of 82% of the ORFs belonging to bacteria and approximately 18% of the ORFs originating from unclassified organisms, in all the samples (Additional file 1: Supplementary Figure S1b). At phylum level, we observed, in total, 103 bacterial groups, 6 fungal groups, and 11 archaeal groups in all the samples. *Acidobacteria* (20.1–25.3% of the sequences) was the most abundant phylum in unamended and ^12^C-EPS-amended control samples, while *Actinobacteria* (26% of the sequences) was the predominant group in “heavy” fraction samples (Additional file 1: Supplementary Figure S1b). At genus level, we found 1541 groups, of which 667 were unclassified. The top three most abundant groups in both control treatments were “unclassified microorganisms” (17.3–19.4% of the ORFs), “unclassified *Bacteria*” (12.7–16% of the ORFs), and “unclassified *Acidobacteriaceae*” (9.3–11.5%), while the predominant groups in “heavy” fraction samples were “unclassified microorganisms” (16.1% of the ORFs), “unclassified *Bacteria*” (18.9% of the ORFs), and “unclassified *Planctomycetes*” (9% of the ORFs) (Fig. 1b).

**Fig. 2:**
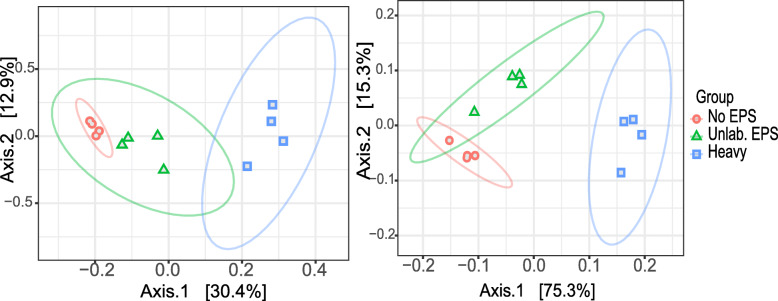
Principal Coordinate Analysis (PCoA) clustering of normalized and Hellinger-transformed SIP metagenome sequencing data based on Bray-Curtis distances of **a** SSU rRNA gene taxonomic classification and **b** ORF taxonomic classification. No EPS: incubation without WH15EPS. Unlab.: EPS-incubation containing ^12^C-WH15EPS. Heavy: “heavy fraction” of incubations containing ^13^C-WH15EPS

PERMANOVA (*p* values < 0.001) showed that, for both SSU rRNA data and ORF-based analysis, the microbial communities were different between treatments, with both control treatments closer to each other, and “heavy” fraction samples separated from both control treatments in PCoA graphs (Fig. 2). For SSU rRNA communities, the first two axes of PCoA explained 43.3% of the variation, while for ORF based data, 90.6% of the variation was explained. RDA analysis (*p* = 0.002) for both datasets showed that mainly groups of *Planctomycetes*, such as “unclassified *Planctomycetes*”, “unclassified *Planctomycetales*,” “unclassified *Planctomycetia*” and *Singulisphaera*, were driving the dispersion of the microbial communities between “heavy” fraction and both control treatments (Additional file 1: Supplementary Figure S2), consistently with the higher abundance of *Planctomycetes* in labeled samples. Alpha diversity indices showed that richness and diversity indices were lower for “heavy” fraction samples in comparison with both controls (Additional file 1: Supplementary Figure S3), supported by ANOVA test (*p* value < 0.05).

#### Functional profile of SIP metagenome

**Fig. 3:**
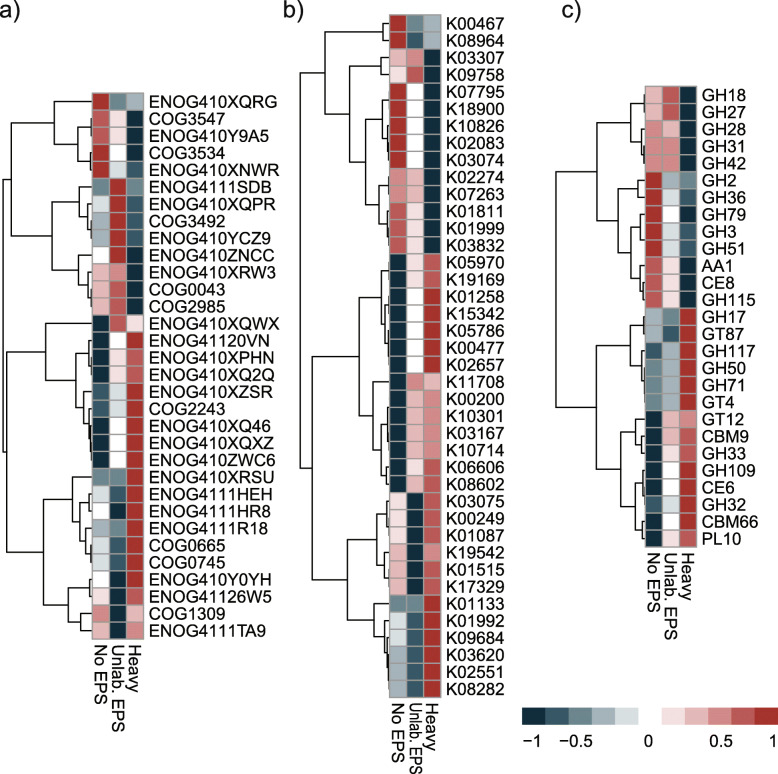
Boruta random forest feature selection of functions that significantly segregated across treatments based on 1000 permutations for **a** COG annotation, **b** KEGG annotation, and **c** dbCAN annotation. Heatmaps based on the *z*-scored TPM normalized relative abundances of annotated ORFs from SIP metagenome samples. The description of the functions displayed in the heatmap is detailed in Supplementary Table S1 (COG), Supplementary Table S2 (KEGG), and Supplementary Table S3 (dbCAN). No EPS: incubation without WH15EPS. Unlab.: EPS-incubation containing ^12^C-WH15EPS. Heavy: “heavy fraction” of incubations containing ^13^C-WH15EPS

KEGG, COG, and CAZy databases were employed for functional gene annotation to explore the functional characteristics of the microbial communities. Approximately 60% of the ORFs were assigned to COGs, matching in total to 20,644 COGs. The most abundant COG categories in all the samples were “R-general function prediction” (10.8–11.6% of the ORFs) (Additional file 1: Supplementary Figure S4a). Boruta feature selection “random forest” analysis (*p* < 0.05) was used to identify feature annotations that segregated significantly between treatments. A total of 32 COGs were selected by Boruta algorithm. Thirteen among the identified COGs were more abundant in the unamended control samples, while 19 were more abundant in the labeled samples (Fig. 3a). However, most of the features identified by the analysis belonged to the category unknown function. Some of the unknown COGs abundant in the labeled treatment, though, were associated mostly to phyla *Planctomycetes* and *Acidobacteria*, according to eggNOG database v 4.5 (Additional file 1: Supplementary Table S1).

KEGG analysis demonstrated that about 50% of the ORFs were assigned to 7,343 KEGG functional orthologs. The 17 most abundant KEGGs in all samples were assigned to three categories: signaling and cellular processes (8 KEGGs—0.16% of the total ORFs), genetic information and processing (6 KEGGs—0.14% of the total ORFs), and metabolism (3—0.21% of the total ORFs) (Additional file 1: Supplementary Figure S4b). Boruta feature selection identified 40 KEGGs that influenced the dispersion of the samples, of which 26 were more abundant in the labeled treatment and 14 were more abundant in the unamended control (Fig. 3b). Among the KEGGs more abundant in the labeled treatment, 13 could be assigned to KEGG pathways, mostly related to “metabolic pathways” and “microbial metabolism in diverse environments” (Additional file 1: Supplementary Table S2). Within the KEGGs more abundant in the unamended control treatment, 8 could be assigned to KEGG pathways, the majority related to “metabolic pathways” (Fig. 3b, Additional file 1: Supplementary Table S2).

Annotation using dbCAN database showed that families GT41 (8.4–11% of the CAZYmes), AA3 (4.4–5%), GT4 (3.4–4.7%), GT2 (4.1–4.3%), and CE10 (3.5–4.2%) were among the most predominant in all the treatments (Additional file 1: Supplementary Figure S4c). Boruta feature selection identified 27 CAZY families affecting the dispersion of the sample treatments (Fig. 3c), the vast majority belonging to the category glycoside hydrolase (GH). Among the selected families, 15 were more abundant in the labeled treatment, and 12 were more abundant in the unamended control. The categories abundant in the labeled treatment involved xylan and fructan modules, xylanases, mannosyltransferases, and agarases, while the categories abundant in the unamended controls were mostly α and β galactosidases and glucosidases (Additional file 1: Supplementary Table S3). PERMANOVA (*p* values < 0.001) demonstrated that for KEGG, COG, and dbCAN data, the functional gene compositions were different between treatments, similarly to taxonomic analysis, with control treatments grouping together and separated from “heavy” fraction samples (Additional file 1: Supplementary Figure S5).

### Cultivated microbes metagenome

#### Overview of the metagenomics data

A total of 422,735,048 reads were obtained after sequence quality filtering, with an average of 80% of the ORFs classified with KEGG and COG databases. The sequencing statistics are described in Table 2.

**Table 2:** Cultivated shotgun metagenome sequencing statistics for each plate. Average from 2 replicates per plate

Statistics	Plate 1	Plate 2	Plate 3	Plate 4
Number of reads	49,148,370	54,247,258.5	58,397,852	49,574,043.5
Assembled reads	1.47E+10	1.6224E+10	1.75E+10	1.4796E+10
Number of contigs	67,980,868	76,125,070.5	82,202,170	68,767,888
Number of predicted genes	159,832	254,727	535,677	479,683
KEGG (% classified ORFs)	66.2	67.2	65.2	63.9
COG (% classified ORFs)	94.6	94.5	94.1	94.0
CAZYmes (%)	4.5	4.7	4.7	4.5
GC content (%)	59.6	60.3	59.0	58.2

#### Community composition of cultivated microbes metagenome based on SSU rRNA and ORF classification

**Fig. 4:**
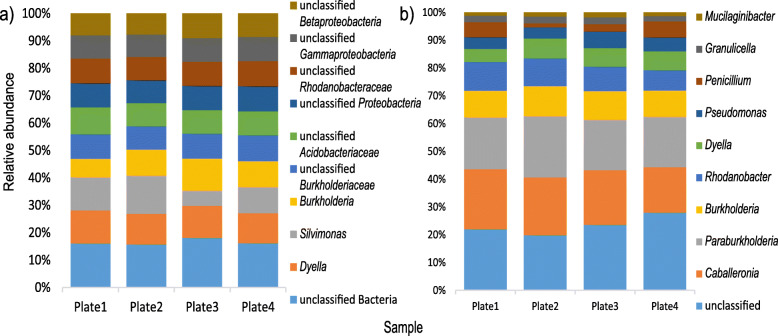
Taxonomic composition and relative abundance of microbial groups at genus level in samples from the metagenome shotgun of cultivated microorganism based on **a** SSU rRNA gene taxonomic classification and **b** ORF taxonomic classification. Only the ten most abundant groups are displayed. Average from 2 replicates per plate of culture medium

Analysis of the taxonomic composition based on SSU rRNA showed an average of 73% of the sequences belonged to domain *Bacteria*, 20% to kingdom *Fungi*, and 7% were derived from other Eukaryotes (Additional file 1: Supplementary Figure S6a). At phylum level, 17 bacterial groups, 7 fungal groups, and 14 eukaryotic groups were identified. The most abundant group was the bacterial phylum *Proteobacteria*, with ~ 47.9% of the sequences, followed by fungal phylum *Ascomycota*, with ~ 14.5% of the sequences (Additional file 1: Supplementary Figure S6b). At genus level, 450 groups in total were observed, with the most abundant groups being bacterial groups. The predominant groups were “unclassified *Bacteria”* (~ 2.2% of the sequences) and *Dyella* (~ 1.5% of the sequences) (Fig. 4a). *Silvimonas* and *Burkholderia* were also among the top 10 most abundant genera (~ 1.4 and 1.3% of the sequences, respectively). Similarly, for the ORF based data, the most abundant groups at genus level belonged to domain *Bacteria*, revealing the presence of 1930 groups at genus level. “Unclassified microbes” was the most abundant group, followed by genera *Caballeronia* (15.4% of the ORFs) and *Paraburkholderia* (15.1% of the ORFs) (Fig. 4b). Other genera, such as *Burkholderia*, *Rhodanobacter*, and *Dyella* were also among the predominant groups (7.8, 7.1, and 4.9% of the ORFs) (Fig. 4b).

#### Functional profile of cultivated microbes metagenome

**Fig. 5:**
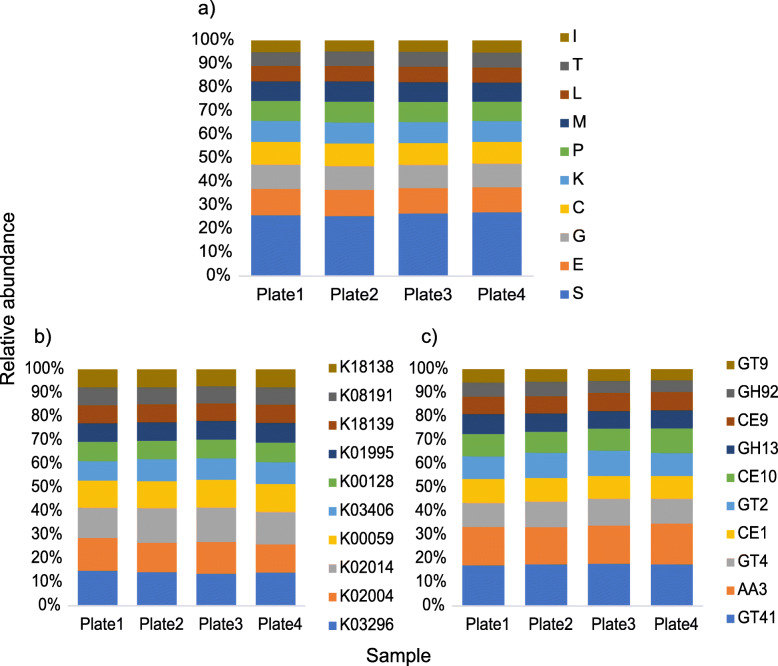
Relative abundance distribution of the most abundant functional categories in TMM-normalized metagenome sequencing data from the shotgun metagenome of cultivated microorganisms. **a** COG annotation (10 most abundant ). **b** KEGG annotation (10 most abundant). **c** dbCAN annotation (10 most abundant). The descriptions of the functions displayed in **b** and **c** are detailed in Supplementary Table S4. Average from 2 replicates per plate of culture medium. E-amino acid transport and metabolism; G-carbohydrate transport and metabolism; H-coenzyme transport and metabolism; C-energy production and conversion; I-lipid transport and metabolism; F-nucleotide transport and metabolism; Q-secondary metabolites; D-cell cycle; N-cell motility; M-cell wall/membrane/envelope biogenesis; V-defense mechanisms; P-inorganic ion transport and metabolism; U-intracellular trafficking; O-post translational modification; T-signal transduction mechanisms; L-replication, recombination, and repair; K-transcription; J-translation; S-function unknown; R-general function and prediction; X-mobilome

The functional profile of the cultivated microbes’ metagenome was explored through the annotation with KEGG, COG, and dbCAN databases. COG analysis demonstrated that approximately 20.6% of the annotated COGs were assigned to unknown functions. Among the classified COGs, similarly to SIP metagenome, the predominant categories involved “E-amino acid transport and metabolism” (~ 8.6% of the ORFs), “G-carbohydrate transport and metabolism” (~ 8.0% of the ORFs), and “C-energy production and conversion” (~ 7.3% of the ORFs) (Fig. 5a).

KEGG pathway analysis showed that around 65% of the ORFs were assigned to 9945 KEGG orthologs. The 20 most abundant KEGGs were distributed in the categories “Genetic information processing” (1 KEGG ~ 0.24% of the total ORFs), “Metabolism” (4 KEGGs ~ 1.18% of the total ORFs), and “Signaling and cellular processes” (15 KEGGs − 4.54% of the ORFs), of which 13 KEGGs were classified as transporters (Fig. 5b).

The analysis of the carbohydrate-active enzymes with dbCAN demonstrated the presence of 298 CAZyme families. Twenty-three families were predominant, which abundance was above 1%. Within the most abundant families, we observed 2 AA families (7.75% of the CAZymes), 1 CBM family, 4 CE families, 10 GH families, and 6 GT families (Fig. 5c). Those CAZyme families comprise mostly enzymes with cellulolytic (alpha-glucosidases, alpha-fucosidases), hemicellulolytic (alpha-rhamnosidases, alpha-xylosidases, alpha-mannosidases, beta-galactosidases), and cell wall metabolism activities (N-acetylglucosaminyltransferases, alpha-N-acetylgalactosaminidases, and peptidoglycan lyases) (Additional file 1: Supplementary Table S4). The most abundant family was GT41 (Fig. 5c), which encompasses UDP-GlcNAc: peptide β-N-acetylglucosaminyltransferases and UDP-Glc: peptide N-β-glucosyltransferases, enzymes involved in protein glycosilation. Among the GH families, the most abundant was GH13.

Among all 127 GH families found in both metagenome datasets, 114 families were observed in both datasets, while 5 families were exclusive from the SIP dataset (GH112, GH48, GH52, GH86, GH98) and 8 were exclusive from the cultivated microbes dataset (GH111, GH131, GH132, GH134, GH45, GH7, GH80, GH85) (Additional file 1: Supplementary Figure S7).

#### Taxonomy of the enriched glycoside hydrolase families

Taxonomic analysis of the most abundant GH family in both metagenome datasets, GH13, demonstrated that the majority of the sequences of GH13 in the cultivated microbes dataset belonged to phyla *Proteobacteria* (66.8% of the GH sequences) and *Acidobacteria* (21.8% of the GH sequences), while in the SIP dataset the most abundant phyla for GH13 were *Actinobacteria* (20.4–45.7% of the GH sequences), *Acidobacteria* (4–24.7% of the sequences), and other phyla (27–34% of the GH sequences) (Table 3).

**Table 3:** Taxonomy associated to sequences of glycoside hydrolases belonging to GH13 family (most abundant) and the enriched GH families in heavy fraction samples from SIP metagenome

**GH families**	**Sample**	***Proteobacteria***	***Acidobacteria***	***Actinobacteria***	***Planctomycetes***	**Others**
GH13	Cultivated	66.8 (2605)	21.8 (827)	0.9 (36)	0.09 (1)	10.4 (51.4)
GH13_SIP	Control	20.1 (128)	23.4 (148)	25.6 (162)	0.1 (4)	31 (196)
	EPS	17.9 (89)	24.7 (125)	20.4 (95)	2.9 (13)	34 (172)
	Labeled	17.2 (127)	4 (30)	45.7 (333)	5.7 (42)	27 (201)
GH109	Control	11 (22)	45 (92)	12 (26)	2 (4)	31 (68)
	EPS	7 (19)	26 (76)	7 (18)	21 (60.8)	40 (111.6)
	Labeled	7 (34)	9 (48)	13 (62)	29 (150)	42 (217)
GH117	Control	0 (0)	33 (1)	33 (1)	0 (0)	33 (1)
	EPS	0 (0)	0 (0)	17 (1)	0 (0)	38 (5)
	Labeled	9 (1)	0 (0)	27 (3)	0 (0)	64 (7)
GH50	Control	100 (3)	0 (0)	0 (0)	0 (0)	0 (0)
	EPS	100 (2)	0 (0)	0 (0)	0 (0)	0 (0)
	Labeled	8 (2)	0 (0)	0 (0)	0 (0)	92 (24)
GH32	Control	0 (0)	44 (4)	11 (1)	0 (0)	44 (4)
	EPS	0 (0)	11 (2)	5 (1)	5 (1)	79 (15)
	Labeled	6 (2)	14 (5)	3 (1)	9 (3)	69 (24)
GH17	Control	75 (9)	0 (0)	0 (0)	0 (0)	25 (3)
	EPS	43 (3)	0 (0)	0 (0)	0 (0)	57 (4)
	Labeled	44 (8)	0 (0)	0 (0)	0 (0)	56 (10)
GH71	Control	0 (0)	0 (0)	100 (1)	0 (0)	0 (0)
	EPS	0 (0)	25 (1)	50 (2)	0 (0)	25 (1)
	Labeled	22 (5)	0 (0)	35 (8)	0 (0)	43 (10)

Average percentage from 4 replicates (total number of sequences)

Within GH families that were more abundant in the SIP “heavy” fraction (Fig. 3c), sequences of GH109 belonged mainly to *Acidobacteria* (45% of the GH sequences), other phyla (31–42% of the GH sequences), and *Planctomycetes* (2–29% of the GH sequences). GH117 family sequences belonged predominantly to *Actinobacteria* (17–33% of the sequences), *Acidobacteria* (0–33% of the GH sequences), and other phyla (33–64% of the GH sequences). Family GH50 sequences belonged mainly to *Proteobacteria* (8–100% of the GH sequences) and other phyla (0–92% of the GH sequences). GH 32 sequences were affiliated mainly to *Acidobacteria* (11–44% of the GH sequences) and other phyla (44–79% of the GH sequences). GH17 sequences belonged to phylum *Proteobacteria* (44–75% of the GH sequences) and other phyla (25–57% of the GH sequences). GH71 sequences were affiliated to phyla *Actinobacteria* (35–100% of the GH sequences), *Proteobacteria* (0–25% of the sequences), *Acidobacteria* (0–25% of the sequences), and other phyla (0–43% of the sequences).

#### Metagenome-assembled genomes (MAGs) assembled from the cultivated microbes metagenome

The binning process using contigs longer than 5 kb generated, after curation and quality filtering, 4 draft genomes. The genome length ranged from 3.0 to 6.3 Mb, and the GC content ranged from 57 to 62%. All MAGs belonged to phylum *Proteobacteria*. None of the MAGs was classified to genus level; however, the genomes were closer to genera *Paraburkholderia* (MAG1) and *Amantichitinum* (MAG2 and MAG4). MAG3 closest classification was to family *Rhodanobacteraceae*. The characteristics of the genomes are described in Table 4. The coverage of the genomes is described in Additional file 1: Supplementary Table S5.

**Table 4:** Genome characteristics for the 4 metagenome-assembled genomes (MAGs) obtained in this study

Genome	MAG1	MAG2	MAG3	MAG4
Taxonomy (closest hit)	*Burkholderiaceae* 95% (*Paraburkholderia* 86%)	*Neisseriaceae* 42% (*Amantichitinum*: 42%)	*Rhodanobacteraceae* 77%	*Neisseriaceae*: 42% (*Amantichitinum* 42%)
Length (Mb)	6.3	3.0	4.8	3.7
Contigs	1997	997	80	1482
Completeness (%)	83.2	79.6	99.7	87.5
Contamination (%)	4.76	3.92	2.44	5
GC (%)	62	57	59	57
Number of predicted genes	7126	3580	4280	4552
Hits to protein database				
KEGG %	90.1	96.8	79.2	93.8
COG %	85.3	85.1	80.2	84
DBcan *n* (%)	279 (3.9)	141 (3.9)	210 (4.9)	180 (4.0)

**Fig. 6:**
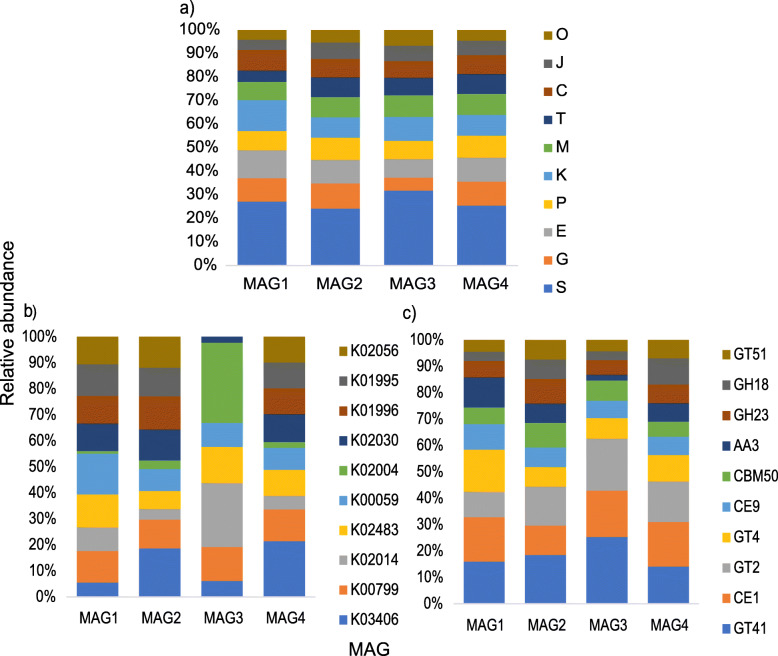
Relative abundance distribution of the most abundant functional categories in metagenome assembled genomes (MAGs) assembled from the shotgun metagenome of cultivated microorganisms sequencing data. **a** COG annotation (10 most abundant). **b** KEGG annotation (10 most abundant). **c** dbCAN annotation (10 most abundant). The description of the functions displayed in **b** and **c** are detailed in Supplementary Table S6 and Supplementary Table S11, respectively. E-amino acid transport and metabolism; G-carbohydrate transport and metabolism; H-coenzyme transport and metabolism; C-energy production and conversion; I-lipid transport and metabolism; F-nucleotide transport and metabolism; Q-secondary metabolites; D-cell cycle; N-cell motility; M-cell wall/membrane/envelope biogenesis; V-defense mechanisms; P-inorganic ion transport and metabolism; U-intracellular trafficking; O-post translational modification; T-signal transduction mechanisms; L-replication, recombination, and repair; K-transcription; J-translation; S-function unknown; R-general function and prediction; X-mobilome

Approximately, 83.7% of the ORFs predicted for the MAGs could be assigned to COGs. The analysis showed that most of the COG assigned ORFs fell on the category “S-function unknown” (16.4–18.4% of the ORFs). Among the classified COGs, however, the most abundant categories were “K-transcription” (5.9–9% of the ORFs), “E-amino acid metabolism” (4.8–8.1% of the ORFs), “G-carbohydrate metabolism” (3.32–7.2%), “C-energy production” (4.2–5.9%), “P-inorganic ion metabolism” (4.65–6.3%), and “M-cell wall/membrane biogenesis” (5.2–5.9%) (Fig. 6a).

KEGG pathway analysis demonstrated that around 90% of the predicted ORFs could be assigned to KEGG orthologs. The majority of the most abundant KEGG orthologs in all the MAGs were related to several types of transporter functions (Fig. 6b and Additional file 1: Supplementary Table S6). In order to evaluate the features of the MAGs that could be involved in the uptake of the WH15EPS sugar units, we decided to look deeper into the transporters. Twenty-four of the KEGG orthologs observed in MAG1 genome were associated to the transport of several sugars, such as sorbitol, ribose, arabinose, xylose, fructose, rhamnose, glucose, mannose, and multiple sugars (Additional file 1: Supplementary Table S7). Among the KEGG orthologs observed in MAG 2 genome, 62 were related to sugar transport, such as maltose, raffinose, lactose, glucosides, cellobiose, xylose, fructose, rhamnose, glucose, mannose, and multiple sugars (Additional file 1: Supplementary Table S8). MAG3 did not exhibit sugar specific transporters within the 60 KEGGs related to transport function; however, we observed some general type transporters (Additional file 1: Supplementary Table S9). In MAG4, 61 KEGG orthologs related to sugar transport were observed, such as maltose, raffinose, lactose, sorbitol, cellobiose, arabinose, xylose, fructose, rhamnose, glucose, mannose, and multiple sugars (Additional file 1: Supplementary Table S10). We also performed the analysis of the CAZYmes with dbCAN database, in order to find enzymes that could be in associated the breakdown of the WH15EPS. MAG1 possessed 279 CAZymes distributed in 90 families, of which the most abundant were CE1, GT4, GT42, CE10, and AA3 (Fig. 6c). The seventy-six glycoside hydrolases observed were distributed in 43 families, including a wide range of activities, such as endo and exo-mannosidases, alpha- and beta-glucosidases and galactosidases, xylosidases, fucosidases, and rhamnosidases (Additional file 1: Supplementary Table S11). MAG2 possessed 141 CAZymes distributed in 65 families, and GT41, GT2, and CE1 were the most abundant families (Fig. 6c). A total of 51 glycoside hydrolases from 30 families were observed, with activities such as alpha- and beta-glucosidases, beta-galactosidases, mannanases and mannosidases, xylanases, and polygalacturonases (Additional file 1: Supplementary Table S11). In MAG3, 210 cazymes distributed in 81 families were observed, and GT41, GT2, CE1, and CE10 were the most abundant (Fig. 6c). Sixty-four glycosil-hidrolases distributed in 37 families were detected. The activities included alpha- and beta-galactosidases, alpha-glucosidases, mannosidases, mannanases, rhamnosidases, arabinosidades, chitinases, and trehalases (Additional file 1: Supplementary Table S11). The genome of MAG4 displayed 180 CAZymes distributed in 73 families, of which the most abundant were CE1, GT2, and GT41 (Fig. 6c). The 64 glycoside hidrolases were spread among 34 families, including activities such as chitinases, arabinofuranosidases, alpha- and beta-glycosidases, mannosidases, cellulases, xylanases, and polygaracturonases (Additional file 1: Supplementary Table S11). The distribution of most abundant CAZYmes and GH families in both metagenomics datasets and MAGs is depicted in Additional file 1: Supplementary Figure S8.

## Discussion

In the present study, we applied culture-independent and culture-dependent techniques to evaluate microbial diversity and functions involved in the degradation of a microbial biopolymer, WH15EPS, focusing on enzymes of biotechnological interest. First, we compared the functional potential of the environment with and without the presence of WH15EPS, evaluating the taxonomic and functional enrichment produced by the addition of the biopolymer using stable isotope probing (SIP). Second, we used metagenomics to evaluate the functional potential of the microorganisms grown in culture medium with WH15EPS as the sole carbon source.

SIP analysis demonstrated that in both 16S rRNA-metagenome dataset extracted and ORF based characterization, phyla *Proteobacteria*, *Actinobacteria*, *Acidobacteria*, and *Planctomycetes* were the most abundant in WH15EPS amended and unamended treatments. However, the addition of WH15EPS to the litter samples promoted an increase in the abundance of the phylum *Planctomycetes*, which was more evident in “heavy” fraction samples, showing that *Planctomycetes* also play an active part in the degradation of WH15EPS. Furthermore, at genus level in the 16S rRNA based analysis, “unclassified *Planctomycetes*” and *Singulisphaera*, which belong to the same phylum, were the most abundant groups in the labeled treatment, while “unclassified *Planctomycetes*” was also among the most abundant in the ORF-based analysis. *Proteobacteria*, *Actinobacteria*, and *Acidobacteria* are widely known to be involved in carbon-degradation processes, for instance, glucose [29], xylan [30], and cellulose assimilation [31]. The glycolytic potential of phylum *Planctomycetes* was recently demonstrated by Ivanova et al. [32], in which genus *Singulisphaera*, for instance, responded significantly to pectin and xylan amendments.

The cultivation-dependent approach demonstrated, as expected, a lower taxonomic diversity, in which the widely studied *Proteobacteria* were among the most abundant. The discrepancy between the diversity of taxa, especially the most abundant groups, observed in cultured and uncultured-based techniques is defined as “The Great Plate Count Anomaly” [33]. The cultivability of microorganisms in laboratory depends of many factors, such as nutrients, oxygen level, temperature, pH, and growing factors [34], limiting the total assortment of taxa that can be actually recovered in culture media. Nevertheless, adding WH15EPS as an alternative carbon source allowed us to demonstrate that several still unknown microorganisms can be grown in laboratorial conditions if unusual compounds are explored. The lower diversity in the culture media plates permitted the assembly of 4 draft genomes related to the most abundant *Proteobacteria*, which classification until genus level was not possible, once more demonstrating the enrichment and potential for isolation of previously unknown microbes.

In order to find potential enzymes of biotechnological interest, we investigated the diversity of CAZymes in both culture-independent and culture-dependent generated datasets, due to their importance in almost all industrial sectors, such as chemical, pharmaceutical, and food industries, as well as production of detergents, textiles, leather, paper, and bioenergy [4]. Furthermore, we also investigated the presence of enzymes that could be employed for biofilm removal.

Among all CAZymes observed, the most abundant families belonged to glycoside transferase families, such as GT41, GT2, and GT4, either in culture-based or in culture-independent datasets. GTs are known to catalyze the formation of glycosidic bonds by transferring a sugar residue from a donor to an acceptor, which could be carbohydrates, proteins, lipids, DNA, and other molecules [35]. Even though a large proportion of genes of microorganism’s genomes in general encode for GTs (about 1–2% of the total number of genes) [36], those enzymes are still not as well explored as GHs [35]. Glycosilated compounds play a wide range of roles, such as energy storage, cell integrity and signaling, among others, and the glycosilation of natural products is important in the exploration of bioactive compounds [37]. GTs are involved in the production of antibiotics, such as chloroeremomycin [38], vancomycin [39], and erythromycin D [40]; therefore, they might be of interest especially for the pharmaceutical industry.

Within glycoside hydrolases, the most abundant family in both metagenomics datasets was GH13 (from *Proteobacteria*), which encompasses starch and pullulan modifying enzymes, including α-amylases, pullulanases, α-1,6-glucosidases, branching enzymes, maltogenic amylases, neopullulanases, and cyclodextrinases [41]. Amylases are among the most important enzymes for food industry, where they can be employed for production of glucose and maltose syrups, reduction of viscosity of syrups, production of clarified fruit juices, solubilization of starch for brewing processes, and manufacture of baked products [12]. Furthermore, the application of α-amylases for the inhibition of biofilm formation has been investigated. In the study of Fleming et al. [42], the use of amylase (from *Bacillus subtilis*) and cellulose (from *Aspergillus niger*) solutions to biofilms of *S. aureus* and *P. aeruginosa* decreased biomass significantly, increasing the effectiveness of antibiotics treatments. A similar effect was observed in the study of Craigen et al. [43], where a commercially available α-amylase detached the aggregates produced by *S*. *aureus* and inhibited biofilm production.

Notwithstanding, feature selection with Boruta package revealed the differential abundance of GH families in “heavy” fraction SIP samples, originated from microorganisms that are believed to be able to degrade WH15EPS. These microorganisms belonged mainly to phyla *Proteobacteria*, *Acidobacteria*, *Actinobacteria*, *Planctomycetes*, as well as high proportion of unknown microorganisms. GH109 (*Acidobacteria* and *Planctomycetes*) contains α-N-acetylgalactosaminidases, which might be employed in the development of universal red blood cells, through the enzymatic removal of monosaccharides from red blood cells’ membranes, and improvement of blood supply in hospitals [44]. Furthermore, those enzymes can be involved in the deconstruction of WH15EPS, since it contains units of xylose, glucose, and arabinose [23]. Families GH117 (*Acidobacteria* and *Actinobacteria*) and GH50 (*Proteobacteria*) contain agarases, which can be used for the production of oligosaccharides with antioxidant activities for applications in food, pharmaceutical, and cosmetic industries [45]. Family GH32 (*Acidobacteria*) comprises invertases and inulinases, enzymes that can be applied in food and fermentation processes [46, 47]. GH17 (*Proteobacteria*) is composed of endoglucanases with activity against β-glucan and laminarin, effective additives for the degradation of polysaccharides for animal feed [47]. Mutanases belonging to GH71 (*Actinobacteria*) family already showed activity against glucans present in dental plaque [48].

Interestingly, sixteen of the most abundant GH families in the culture-independent dataset were found to be the predominant in the culture-dependent approach, and all the GH families with higher abundances in the labeled SIP samples were also observed in the culture-dependent dataset. Furthermore, the MAGs also contained GH families of interest, with variable abundances among them. MAG1 (similar to *Paraburkholderia*) contained 8 ORFs belonging to family GH92, which encompasses alpha-mannosidases with applications in food and pharmaceutical industries, for the production of juices, degradation of plant material, or coffee extraction [49]. In MAG2 (similar to *Amantichitinum*), five ORFs were classified as GH23, which contains lysozymes that can be used as polysaccharide hydrolysers for biofilm breakdown [2, 50]. MAG3 (*Rhodanobacteraceae*) is abundant in GH92 and GH23 but also GH2 family ORFs, which comprises several enzymes. Within the best characterized ones, there are β-galactosidases employed for the production of lactose-free milk products and other galactooligosaccharides [51]. MAG4 (similar to *Amantichitinum*) is rich in GH18 enzymes, involving chitinases that for instance are important agents with applications for fungal biological control and bioremediation processes [52]. It is important to recognize that, even though the MAGs possessed a low level of contamination (< 5%), they do not represent genomes of axenic cultures from isolated microorganisms. Therefore, the corresponding laboratory cultures should still be recovered in order to fully validate our MAGs.

Our study showed that, using SIP and a complex EPS (WH15EPS), we could detect the subset of the total microbial community that was capable of incorporating the biopolymer. Among those we observed members of *Planctomycetes* as an interesting target for biotechnological studies and heterologous expression, which could be performed also in several other genes, combining bioinformatics, gene synthesis, and enzymatic screening [53]. In addition, we demonstrated that functional diversity induced by the presence of WH15EPS in both culture-dependent and culture-independent approaches was enriched in genes coding for GHs, for instance, amylases, chitinases, agarases, and endoglucanases and that could be applied in chemical, pharmaceutical, and food industries. Furthermore, the use of WH15EPS may be employed for the investigation and isolation of yet unknown taxa, such as unclassified *Proteobacteria* and *Planctomycetes*, increasing the number of current cultured bacterial representatives.

## Conclusions

We observed, in the functional diversity induced by the presence of WH15EPS in both culture-dependent and culture-independent approaches, the presence of 310 CAZyme families, from which 38.4% (119) were GH families. GHs of biotechnological interest could potentially be employed in almost all industrial sectors, such as chemical, pharmaceutical, and food industries, as well as production of detergents, textiles, leather, paper, and bioenergy. Furthermore, we also observed the presence of enzymes that could be employed for biofilm removal. Even though the potential enzymes might belong to slow growing microorganisms in laboratorial conditions, such as *Acidobacteria*, *Planctomycetes*, and *Verrucomicrobia*, sequences can still be targeted for further heterologous expression and characterization. In addition, the culture-based metagenomics dataset allowed the assembly of 4 metagenome-assembled genomes (MAGs) that potentially belong to unclassified *Proteobacteria.* We showed that WH15EPS may be employed for the isolation of known and unknown microbes, as well as the targeting of sequences of a wide range of CAZyme families.

## Material and methods

### Soil samples

**Fig. 7:**
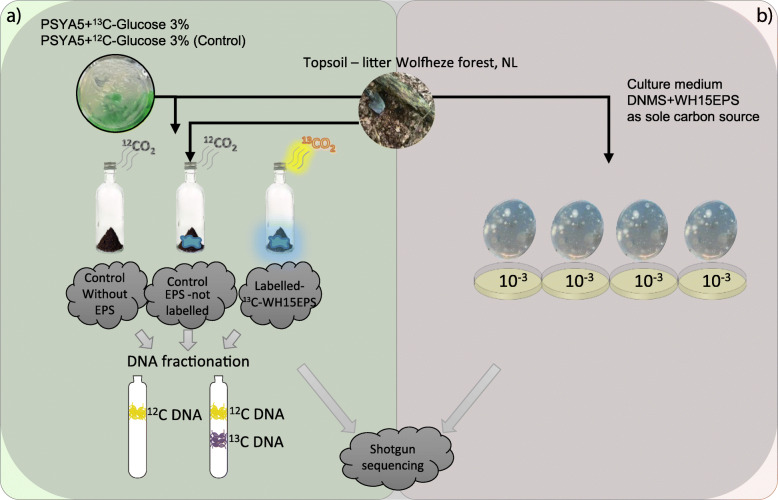
Workflow diagram of the experimental design. **a**^13^C-Glucose and ^12^C-Glucose was used in PSYL5 culture medium for ^13^C- and ^12^C-WH15EPS production by *Granulicella* sp WH15. ^13^C- and ^12^C-WH15EPS were purified and incubated with litter-topsoil samples collected in Wolfheze forest, NL. Controls without WH15EPS were also incubated; each treatment had 6 replicates. After 35 days of incubation and CO_2_ respiration measurements, DNA was extracted and fractionated. “Heavy fraction” of the ^13^C-WH15EPS incubations and total DNA from ^12^C-WH15EPS and controls without EPS were sent for shotgun sequencing. **b** In parallel, purified ^12^C-WH15EPS was used as a carbon source for culture medium DNMS. A 10^−3^ dilution of litter-topsoil samples collected in Wolfheze forest was inoculated in the culture medium and incubated at room temperature for 30 days. Each plate had 2 replicates. Next, cells were scraped from the plates; total DNA was extracted and sent for Shotgun sequencing

Four topsoil-litter mixed samples were collected in the spring of 2017 from the Wolfheze forest in the Netherlands (Additional file 1: Supplementary Table S12). Samples were taken from topsoil (0 to 5 cm) adjacent to fallen tree trunks. The collected samples were pooled, sieved (2-mm mesh), and immediately used for SIP incubation with EPS from *Granulicella* sp. strain WH15 (WH15EPS). The physicochemical properties of the topsoil-litter samples were determined (Eurofins Agro BV, Wageningen, NL) and are presented in Additional file 1: Supplementary Table S13. A workflow diagram of the experiments is depicted in Fig. 7.

### SIP metagenome

#### [^13^C]-labeled and unlabeled EPS production

*Granulicella* sp. strain WH15 was cultivated on PSY5 solid medium [54] containing 3% (wt/vol) fully ^13^C-labeled glucose as the sole carbon source or unlabeled glucose for unlabeled control EPS production. After 30 days of incubation at 20 °C polysaccharide portion of EPS was extracted and purified according to Liu et al. [55]. Sixty microliters of 36.5% formaldehyde was added to each sample and incubated at 4 °C for 1 h. Next, 4 ml of 1 M NaOH was added and incubated at 4 °C for 3 h. After centrifugation at 9000×*g* for 40 min, cell debris in the supernatant were eliminated through filtering (0.2 μm membranes, Millipore) at room temperature, and monosaccharides were removed by dialysis in SnakeSkin™ Dialysis Tubing (3500 Da) (Thermo Fisher Scientific, MA, USA) against demineralized water at 4 °C for 48 h. DNA concentration in the EPS solution was determined in a Qubit fluorometer using a broad-range Quant-iT™ dsDNA Assay Kit (Invitrogen, Carlsbad, CA, USA). EPS protein concentrations were determined by a Pierce™ Modified Lowry Protein Assay Kit (Thermo Fisher Scientific, MA, USA). The total carbohydrate content was estimated by the phenol-sulfuric acid method [56] modified for 96-well plates [57] with glucose as the standard. The EPS solutions were freeze-dried at − 80 °C for 72 h until further processing. The purified EPS contained ~ 400 mg/ml carbohydrates, ~ 1% protein, and undetectable amounts of DNA.

#### Stable isotope probing (SIP) incubation

Freeze-dried EPS was hydrated with 1 ml of Milli-Q sterile water immediately before inoculation in topsoil-litter samples to create a homogeneous distribution. Five grams (wet weight) of topsoil-litter samples with 0.05% (wt/wt) WH15EPS (labeled and unlabeled controls) or without EPS were added to a 120-ml bottle, which was sealed with a butyl rubber stopper and incubated at room temperature (22 °C) in the dark. Each treatment (labeled EPS, unlabeled EPS, and control without EPS) had six replicates. In order to maintain oxic conditions and prevent ^13^CO_2_ cross-feeding, all vials were uncapped and aired every 4 days. The use of WH15EPS by the microbial community was monitored as CO_2_ respiration through gas chromatography (GC) (Trace GC Ultra, Thermo Fisher Scientific, MA, USA), performed daily to monitor the vial headspace CO_2_. For incubations with [^13^C]-labeled EPS, monitoring of the headspace CO_2_^13^C/^12^C ratio was performed via GC combustion isotope ratio mass spectrometry (GC/C/IRMS) (GC IsoLink II™ IRMS System, Thermo Fisher Scientific, MA, USA). CO_2_ emissions throughout the experiment are shown in Additional file 1: Supplementary Figure S9. After 35 days of incubation, 0.5 g of samples were removed from the vials for DNA extraction.

#### DNA extraction and fractionation

DNA was extracted from 250 mg of soil with or without ^13^C-labeled/unlabeled substrates with the PowerSoil® DNA Isolation Kit (MO BIO Laboratories, Inc) according to the manufacturer’s instructions and quantified by a spectrophotometer (NanoDrop™ 2000, Thermo Fisher Scientific, MA, USA). Gradient fractionation was performed according to Neufeld et al. [58]. Two microgram of DNA were combined with CsCl (1.72 g/ml) and gradient buffer (100 mM Tris-HCl pH 8.0, 100 mM KCl, 1 mM EDTA) in an ultracentrifugation tube (PA UltraCrimp 1.8 ml, ThermoFisher Scientific, MA, USA) and ultracentrifuged at 125,395×*g* (Discovery 120SE ultracentrifuge, ThermoFisher Scientific, Massachusetts, USA) under vacuum at 20 °C for 65 h. Gradient fractionation resulted in 18 DNA fractions of approximately 100 μl each, which density was measured with a refractometer (AR200, Reichert Technologies, New York, USA). DNA was precipitated from the CsCl with polyethylene glycol solution (30% PEG6000, 1.6 M NaCl) and glycogen (20 μg/μl), washed with 70% ethanol, and eluted in 30 μl of 10 mM Tris-HCl buffer, pH 8.0. The DNA concentration of each fraction was determined in a Qubit 4 Fluorometer (ThermoFisher Scientific, MA, USA) using a Quant-iT™ dsDNA HS Assay Kit (Invitrogen, Carlsbad, CA, USA). The unlabeled substrate incubations were used as controls to determine the expected position of labeled soil DNA in the CsCl density gradients.

Library preparation and high-throughput shotgun sequencing were performed using the “heavy” DNA fractions pooled within each sample replicate as well as the total DNA of both the ^12^C-EPS-amended and unamended controls. Library preparation and Illumina MiSeq PE250 shotgun sequencing were performed at McGill University and Génome Québec Innovation Centre (Montréal, Québec, Canada). The sequences were deposited in the European Nucleotide Archive (ENA; https://www.ebi.ac.uk/ena) under the accession number PRJEB31257.

### Metagenome of cultivated microorganisms in media with WH15EPS as sole carbon source

For evaluation of the metagenome of microorganisms that were able to grow in culture medium with WH15 EPS as a sole carbon source, 10 g of fresh topsoil-litter sample were mixed with 100 ml of 100 mM MES buffer (2-[N-morpholino]ethanesulphonic acid, 1.95 g/l, pH 5.5), agitated for 30 min at room temperature on a vortex and decanted for 30 min. Dilutions (10^−3^ to 10^−6^) were prepared in sterile MES buffer, and 200 μl of the dilutions were plated in quadruplicate. Diluted culture medium DNMS [MgSO_4_.7H_2_O 0.2 g/l, CaCl_2_.2H_2_O 0.053 g/l, chelated iron solution 0.2 ml/l (ferric III ammonium citrate 0.1 g/100 ml, EDTA 0.2 g/100 ml, HCl 0.3 ml/100 ml) trace element solution SL10 1 ml/L [59], NH_4_Cl 0.1 g/l, agar 20 g/l] with added WH15EPS [23] (0.05%) pH 5.5 and 40 ng/μl (40 mg/l) cicloheximide to prevent growth of fungi was used for plating. To prevent caramelization, the freeze-dried purified WH15EPS was hydrated with Milli-Q water, sterilized by filtration through a 0.2 μm membrane (Millipore), and added to the culture medium after autoclaving. Chelated iron solution and trace element solution SL10 were added after autoclaving and cooling of the culture medium. The plates inoculated with the soil suspension were incubated at room temperature for 1 month. The dilution 10^−3^ was chosen for sequencing. After incubation, colonies were scraped and used for total DNA extraction with PowerSoil® DNA Isolation Kit (MO BIO Laboratories, Inc). Following the first DNA extraction, a second round of DNA extraction was performed for each sample, according to Dimitrov et al. [60]. The total DNA extracted from the plates was used for metagenome shotgun sequencing. Library preparation and Illumina HiSeq XTen sequencing were performed at Genewiz (Suzhou, China). The sequences were deposited in the European Nucleotide Archive (ENA; https://www.ebi.ac.uk/ena) under the accession number PRJEB24069.

### Bioinformatics and statistical analyses of metagenome data

#### SIP metagenome

SIP metagenome sequences were processed using EBI MGnify [61] pipeline and SqueezeMeta [62] pipeline in sequential mode. Briefly, in the SqueezeMeta pipeline, trimming and quality filtering were performed using Trimmomatic [63]; assembly for each sample separately was done using Megahit [64]; Prodigal [65] was used for ORF prediction, and barrnap [66] was employed for rRNA gene sequence retrieval, which were classified using RDP classifier [67]. Diamond [68] software was used for taxonomic classification of the ORFs against Genbank nr database and functional annotation with eggNOG database, for KO and COG numbers [69]. eggNOG-mapper [70] was employed for carbohydrate-active enzymes annotation with against dbCAN [71]. SqueezeMeta script SQM2tables.py was used to compute the average coverage and normalized TPM (transcripts per million) values for information on gene and function abundances. Normalized TPM SqueezeMeta ORF dataset and 16S rRNA gene data recovered from MGNify analysis were used for statistical analyses, performed in RStudio version 1.1.423 running R version 3.5.1 [72]. For the 16S-based analysis, OTUs with less than 1 count across all the samples, chloroplast and mitochondrial sequences were discarded; prior to alpha diversity analyses, the data were rarefied to the size of the smallest sample (175 reads). For both ORF-based and 16S gene-based taxonomy datasets, “Phyloseq” package [73] was used to calculate the number of observed OTUs, Shannon and Inverse Simpson diversity indices, and Chao1 and ACE diversity estimators. Significant differences in the estimators between treatments were evaluated through parametric and non-parametric tests, including ANOVA, Kruskal-Wallis, and Tukey’s HSD tests (package “agricolae”) [74]. Bray-Curtis distance matrices constructed using the Hellinger transformed [75] datasets were used for principal coordinate analysis (PCoA) using the capscale function from the “vegan” package v. 2.4.6 [76]. Group dissimilarities were tested by permutational multivariate analysis of variance (PERMANOVA) using the function Adonis from the “vegan” package. CANOCO (version5) [77] was employed to explore the relationship between sample treatments and taxa abundance through redundancy analysis (RDA) in the Hellinger transformed datasets. The statistical significance (*p* value < 0.05) of eigenvalues and treatment-taxa abundance correlations was tested using Monte Carlo permutation test at 499 permutations, and the top 20 taxa associated with the dispersion of the treatments were displayed in RDA graphs.

In order to identify predicted functions (COG, KEGG, and CAZYmes) responsible for the observed clustering patterns, we performed a feature selection using a “random forest” algorithm using the R package Boruta [78] (1,000 trees, *p* value < 0.05). Boruta tests if the importance of each individual variable is significantly higher that the importance of a random variable by fitting random forest models iteratively until all predictor variables are classified as “confirmed” or “rejected” at the 0.05 alpha level [79]. The heatmaps for relevant features for each function were constructed with pheamap [80] R package, based on *z*-score transformed TPM (transcripts per million) abundances to improve normality and homogeneity of the variances. Sequences were submitted to the European Nucleotide Archive (ENA) and are available under the accession number PRJEB31257.

#### Metagenome analysis for cultivated microorganisms

The DNA of the cultivated microorganisms were shotgun metagenome sequenced, and the sequences were processed using EBI MGnify [61] pipeline and ATLAS (Automatic Tool for Local Assembly Structures) [81] pipeline. For ATLAS, quality filtering was performed using BBDuk2, and cross-assembly was done with Megahit [64]; functional and taxonomic analysis were performed at ORF level for the assembled contigs. Prodigal [65] was used for ORF prediction, and eggNOG database [69] was used for functional annotation (COG and KO numbers) using the DIAMOND software [68]. eggNOG-mapper [70] was used for functional annotation of CAZymes with dbCAN [71]. The Kaiju software [82] was used for ORF taxonomy assignment against NCBI RefSeq database. Custom scripts were used to generate tables containing information of taxonomy and function abundance of the ORFs in all samples. Quality controlled contigs > 1000 kb were used for binning using Concoct [83], Maxbin [84], and Metabat [85]; resulting bins were refined using DAS tool [86], and genome dereplication was performed with dRep [87]. Completeness and contamination of the assembled genomes were checked using CheckM [88], as well as taxonomy assignment. The ORFs of the genomes were predicted using Prodigal [65], and DIAMOND software [68] was used for functional annotation with eggNOG (COG and KO numbers) [69]. The annotation of CAZYmes was performed with eggNOG-mapper [70] against dbCAN [71]. Sequences were submitted to the European Nucleotide Archive (ENA) and are available under the accession number PRJEB24069.

## Supplementary information

**Additional file 1 Supplementary Figure S1:** Taxonomic composition and relative abundance of microbial groups at phylum level in SIP metagenome treatments based on a) SSU rRNA gene sequence classification (>2.2 % abundance) b) ORF taxonomic classification (>0.1% abundance). Average abundances of 4 replicates. Unc: unclassified. No EPS – incubation without WH15EPS. Unlab EPS-incubation containing ^12^C-WH15EPS. Heavy – ‘heavy fraction’ of incubations containing ^13^C-WH15EPS; **Supplementary Figure S2:** Biplot of the Redundancy analysis (RDA) based on normalized and Hellinger-transformed abundances of a) SSU rRNA gene taxonomy classification and b) ORF taxonomic classification. Only the best 20 fitting groups are displayed. Unc: unclassified. No EPS – incubation without WH15EPS. Unlab EPS-incubation containing ^12^C-WH15EPS. Heavy – ‘heavy fraction’ of incubations containing ^13^C-WH15EPS; **Supplementary Figure S3:** Box-plot comparisons of alpha-diversity assessment by richness estimators (number of observed OTUs, Chao1, ACE) and diversity indices (Shannon, Inverse Simpson) for SIP 16S rRNA gene samples. ‘Heavy fraction’ values are significantly lower in comparison with both controls for all comparisons (p-value < 0.05). Comparisons performed across treatments using ANOVA test and Tukey`s HSD post-hoc test. Data rarefied to the minimum sampling depth. Unlab. EPS-incubation containing ^12^C-WH15EPS. Heavy – ‘heavy fraction’ of incubations containing ^13^C-WH15EPS; **Supplementary Figure S4:** Relative abundance distribution of the most abundant functional categories in TPM-normalized metagenome sequencing data from the SIP metagenome. a) COG annotation (all categories); b) KEGG annotation (above 0.1 % abundance); c) dbCAN annotation (above 1% abundance). E-Amino acid transport and metabolism; G- Carbohydrate transport and metabolism; H-Coenzyme transport and metabolism; C-Energy production and conversion; I-Lipid transport and metabolism; F-Nucleotide transport and metabolism; Q- Secondary metabolites; D-Cell acycle; N-Cell motility; M-Cell wall/membrane/envelope biogenesis; V-Defence mechanisms; P-Inorganic ion transport and metabolism; U-Intracellular trafficking; O-Post translational modification; T-Signal transduction mechanisms; L-Replication, recombination and repair; K-Transcription; J-Translation; S-Function unknown; R-General function and prediction; X-Mobilome.; **Supplementary Figure S5:** Principal Coordinate Analysis (PCoA) clustering of normalized and Hellinger-transformed SIP metagenome sequencing data based on Bray-Curtis distances of a) COG annotation, b) KEGG annotation and c) dbCAN annotation. No EPS – incubation without WH15EPS. Unlabeled EPS-incubation containing ^12^C-WH15EPS. Heavy – ‘heavy fraction’ of incubations containing ^13^C-WH15EPS; **Supplementary Figure S6:** Taxonomic composition and relative abundance of microbial groups at a) kingdom and b) phylum level in samples from the metagenome shotgun of cultivated microrganims based SSU rRNA gene taxonomic classification. Average from 2 replicates per plate of culture medium.; **Supplementary Figure S7:** Venn diagram depicting the number of common and unique glycoside hydrolase (GH) families observed in SIP metagenome and metagenome of cultivate microorganisms` datasets; **Supplementary Figure S8:** Distribution of the 20 most abundant CAZyme families in a) SIP metagenome samples (relative abundance, average of 4 replicates); b) metagenome of cultivated microorganisms (relative abundance, average of 2 replicates); c) Metagenome-Assembled Genomes (MAGs) (number of genes), and most abundant glycosyl hydrolases (GH) in d) SIP metagenome samples (relative abundance, average of 4 replicates), e) metagenome of cultivated microorganisms (relative abundance, average of 2 replicates) and f) Metagenome-Assembled Genomes (MAGs) (number of genes); **Supplementary Figure S9:** CO_2_ emission. CO_2_ production during total incubation period. Control: control without EPS; EPS: control containing ^12^C-EPS; Labeled: incubation with ^13^C-EPS; Labeled CO_2_ percentage: ^13^CO_2_ emitted during ^13^C-EPS sample incubation; water: days when samples were hydrated; air: days when samples were aired. **Supplementary Table S1:** COG functions that significantly segregated across treatments selected by Boruta random forests algorithm based on 1000 permutations in the SIP metagenome treatment comparisons; **Supplementary Table S2:** KEGG orthologs that significantly segregated across treatments selected by Boruta random forests algorithm based on 1000 permutations in the SIP metagenome treatment comparisons; **Supplementary Table S3:**CAZyme families that significantly segregated across treatments selected by Boruta random forests algorithm based on 1000 permutations in the SIP metagenome treatment comparisons; **Supplementary Table S4:** Most abundant CAZyme families (above 1% abundance) and most abundant KEGG orthologs (above 0.2% abundance) in the shotgun metagenome of cultivated microorganisms; **Supplementary Table S5:** MAGs coverage in all samples; **Supplementary Table S6:** Most abundant KEGG orthologs in MAGs and their associated functions. A selection of the top 10 most abundant KEGG orthologs in each genome is displayed. Annotation performed using eggNOG database.; **Supplementary Table S7:** Sugar transporters in MAG1 annotated with eggNOG database; **Supplementary Table S8:** Sugar transporters in MAG2 annotated with eggNOG database; **Supplementary Table S9:** General type transporters in MAG3 annotated with eggNOG database; **Supplementary Table S10:** Sugar transporters in MAG4 annotated with eggNOG database; **Supplementary Table S11:** Families of CAZymes observed in the MAGs, number of ORFs and associated functions; **Supplementary Table S12:** Coordinates of the sampling sites; **Supplementary Table S13:** Physicochemical properties of topsoil-litter samples.

## Data Availability

The sequences were deposited in the European Nucleotide Archive (ENA; https://www.ebi.ac.uk/ena) under the accession numbers PRJEB24069 and PRJEB31257.
