# Supplementary material for: Cultivation-independent and cultivation-dependent metagenomes reveal genetic and enzymatic potential of microbial community involved in the degradation of a complex microbial polymer
Source: Microbiome. 2020 Jun 1;8:76. doi: 10.1186/s40168-020-00836-7 (PMC7265232; doi:10.1186/s40168-020-00836-7)
Supplement: Supplementary file 2 — Additional file 1 Supplementary Figure S1: Taxonomic composition and relative abundance of microbial groups at phylum level in SIP metagenome treatments based on a) SSU rRNA gene sequence classification (>2.2 % abundance) b) ORF taxonomic classification (>0.1% abundance). Average abundances of 4 replicates. Unc: unclassified. No EPS – incubation without WH15EPS. Unlab EPS-incubation containing 12C-WH15EPS. Heavy – ‘heavy fraction’ of incubations containing 13C-WH15EPS; Supplementary Figure S2: Biplot of the Redundancy analysis (RDA) based on normalized and Hellinger-transformed abundances of a) SSU rRNA gene taxonomy classification and b) ORF taxonomic classification. Only the best 20 fitting groups are displayed. Unc: unclassified. No EPS – incubation without WH15EPS. Unlab EPS-incubation containing 12C-WH15EPS. Heavy – ‘heavy fraction’ of incubations containing 13C-WH15EPS; Supplementary Figure S3: Box-plot comparisons of alpha-diversity assessment by richness estimators (number of observed OTUs, Chao1, ACE) and diversity indices (Shannon, Inverse Simpson) for SIP 16S rRNA gene samples. ‘Heavy fraction’ values are significantly lower in comparison with both controls for all comparisons (p-value < 0.05). Comparisons performed across treatments using ANOVA test and Tukey`s HSD post-hoc test. Data rarefied to the minimum sampling depth. Unlab. EPS-incubation containing 12C-WH15EPS. Heavy – ‘heavy fraction’ of incubations containing 13C-WH15EPS; Supplementary Figure S4: Relative abundance distribution of the most abundant functional categories in TPM-normalized metagenome sequencing data from the SIP metagenome. a) COG annotation (all categories); b) KEGG annotation (above 0.1 % abundance); c) dbCAN annotation (above 1% abundance). E-Amino acid transport and metabolism; G- Carbohydrate transport and metabolism; H-Coenzyme transport and metabolism; C-Energy production and conversion; I-Lipid transport and metabolism; F-Nucleotide transport and metabolism; Q- Secondary [file 40168_2020_836_MOESM1_ESM.docx]

**Supplementary material**

**Supplementary Figure S1:** Taxonomic composition and relative abundance of microbial groups at phylym level in SIP metagenome treatments based on a) SSU rRNA gene sequence classification (>2.2 % abundance) b) ORF taxonomic classification (>0.1% abundance). Average abundances of 4 replicates. Unc: unclassified. No EPS – incubation without WH15EPS. Unlab EPS-incubation containing ^12^C-WH15EPS. Heavy – ‘heavy fraction’ of incubations containing ^13^C-WH15EPS


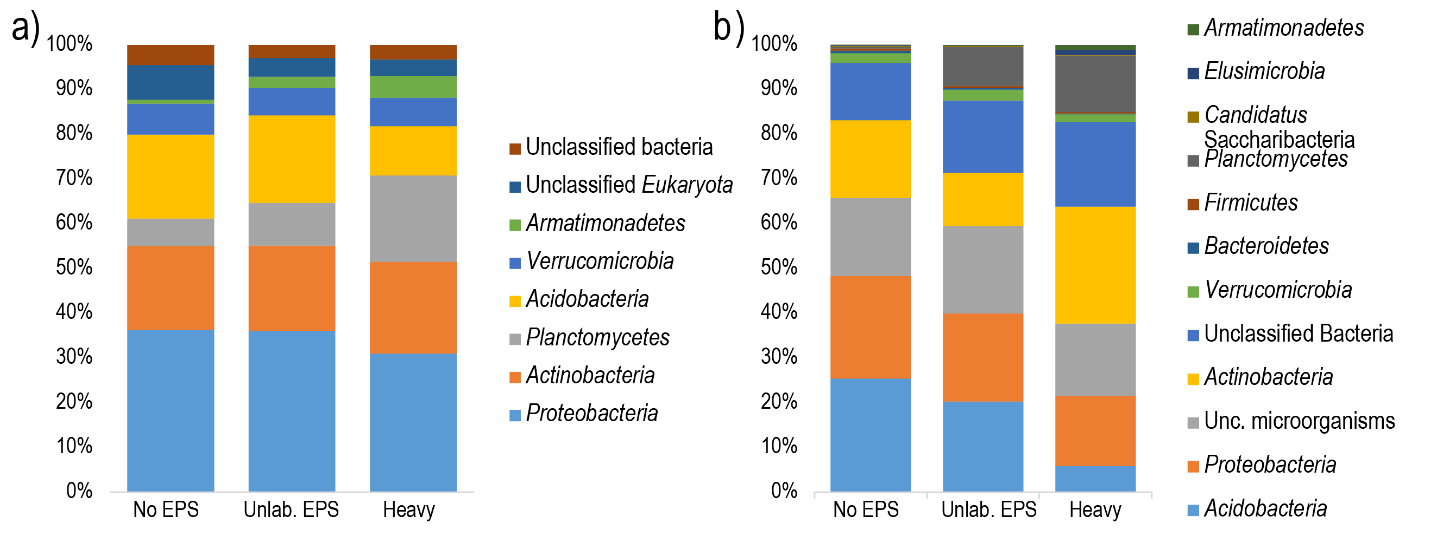


**Supplementary Figure S2:** Biplot of the Redundancy analysis (RDA) based on normalized and Hellinger-transformed abundances of a) SSU rRNA gene taxonomy classification and b) ORF taxonomic classification. Only the best 20 fitting groups are displayed. Unc: unclassified. No EPS – incubation without WH15EPS. Unlab EPS-incubation containing ^12^C-WH15EPS. Heavy – ‘heavy fraction’ of incubations containing ^13^C-WH15EPS


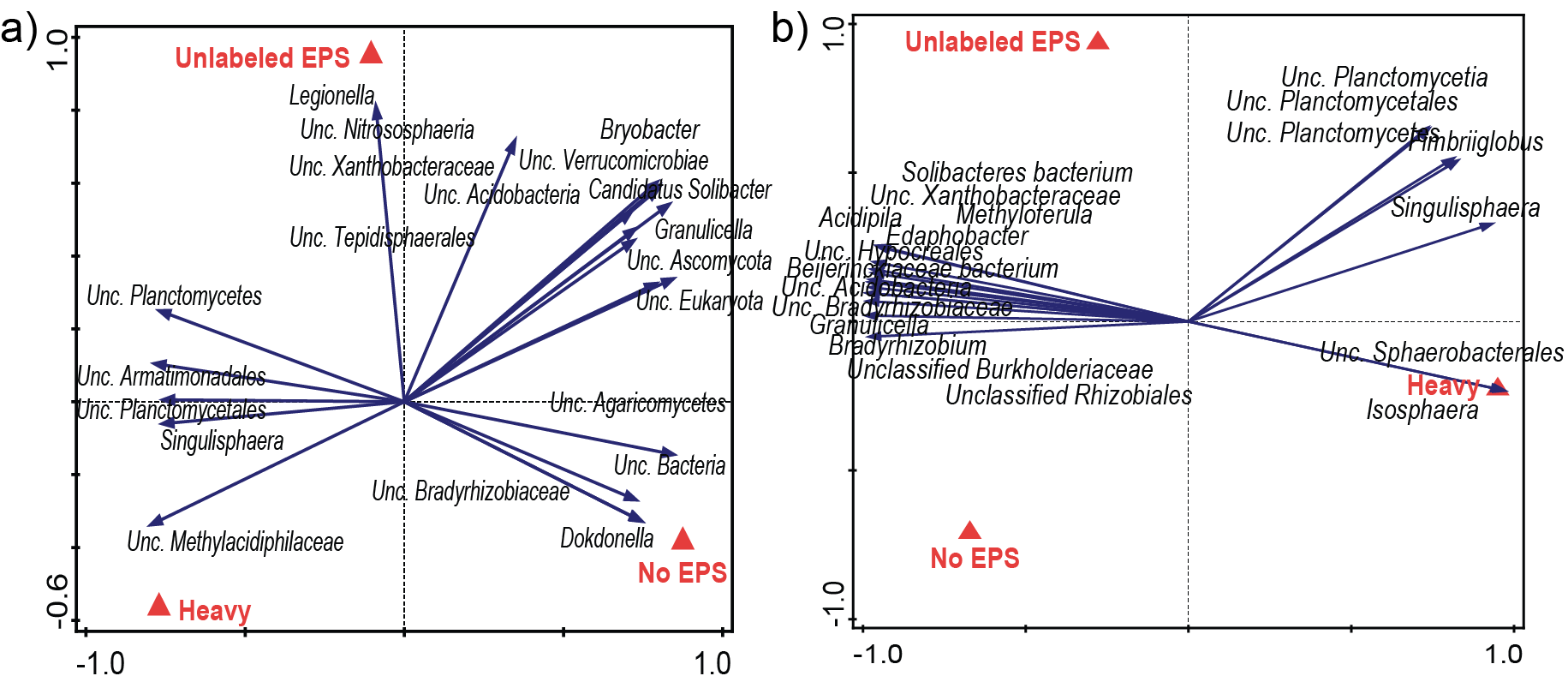


**Supplementary Figure S3:** Box-plot comparisons of alpha-diversity assessment by richness estimators (number of observed OTUs, Chao1, ACE) and diversity indices (Shannon, Inverse Simpson) for SIP 16S rRNA gene samples. ‘Heavy fraction’ values are significantly lower in comparison with both controls for all comparisons (p-value < 0.05). Comparisons performed across treatments using ANOVA test and Tukey`s HSD post-hoc test. Data rarefied to the minimum sampling depth. Unlab. EPS-incubation containing ^12^C-WH15EPS. Heavy – ‘heavy fraction’ of incubations containing ^13^C-WH15EPS.


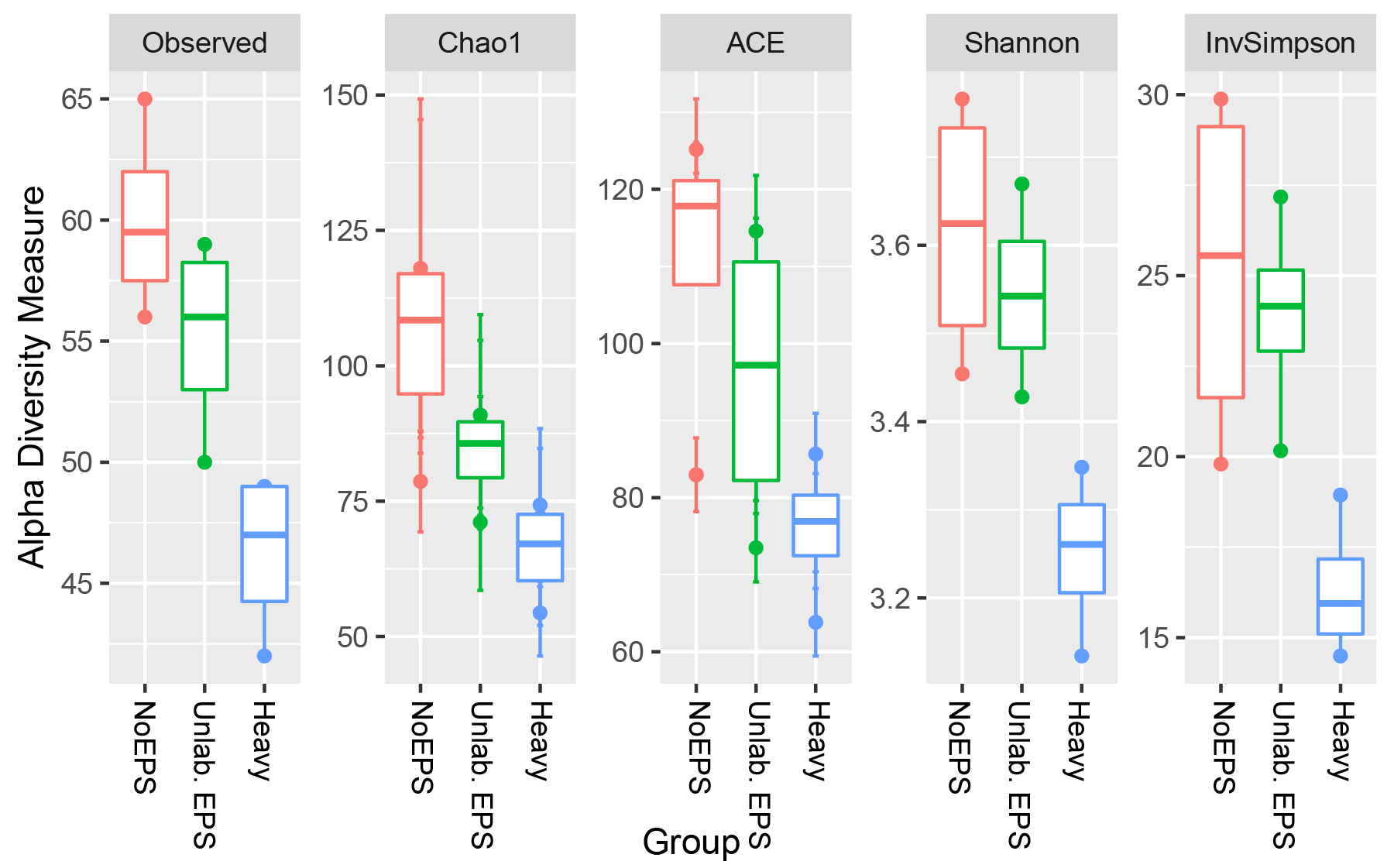


**Supplementary Figure S4:** Relative abundance distribution of the most abundant functional categories in TPM-normalized metagenome sequencing data from the SIP metagenome. a) COG annotation (all categories); b) KEGG annotation (above 0.1 % abundance); c) dbCAN annotation (above 1% abundance). E-Amino acid transport and metabolism; G- Carbohydrate transport and metabolism; H-Coenzyme transport and metabolism; C-Energy production and conversion; I-Lipid transport and metabolism; F-Nucleotide transport and metabolism; Q- Secondary metabolites; D-Cell cycle; N-Cell motility; M-Cell wall/membrane/envelope biogenesis; V-Defence mechanisms; P-Inorganic ion transport and metabolism; U-Intracellular trafficking; O-Post translational modification; T-Signal transduction mechanisms; L-Replication, recombination and repair; K-Transcription; J-Translation; S-Function unknown; R-General function and prediction; X-Mobilome.


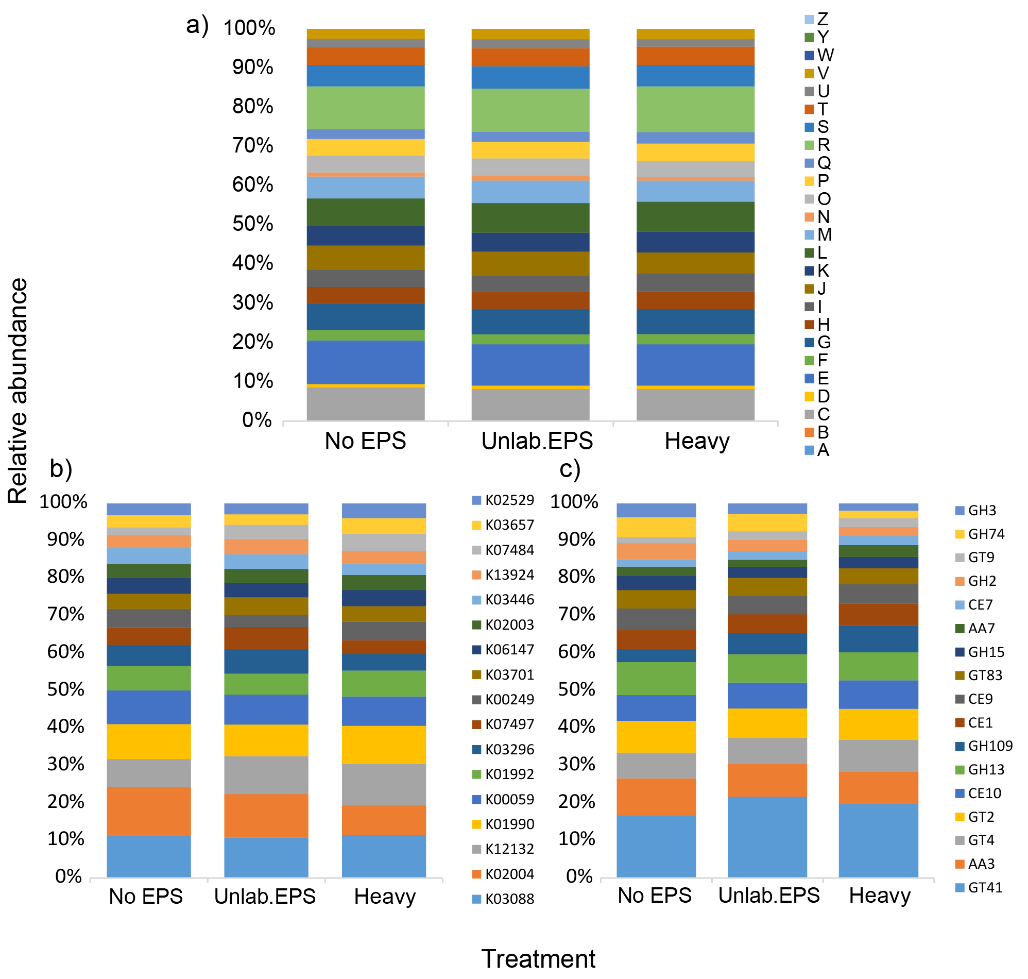


**Supplementary Figure S5:** Principal Coordinate Analysis (PCoA) clustering of normalized and Hellinger-transformed SIP metagenome sequencing data based on Bray-Curtis distances of a) COG annotation, b) KEGG annotation and c) dbCAN annotation. No EPS – incubation without WH15EPS. Unlabeled EPS-incubation containing ^12^C-WH15EPS. Heavy – ‘heavy fraction’ of incubations containing ^13^C-WH15EPS.


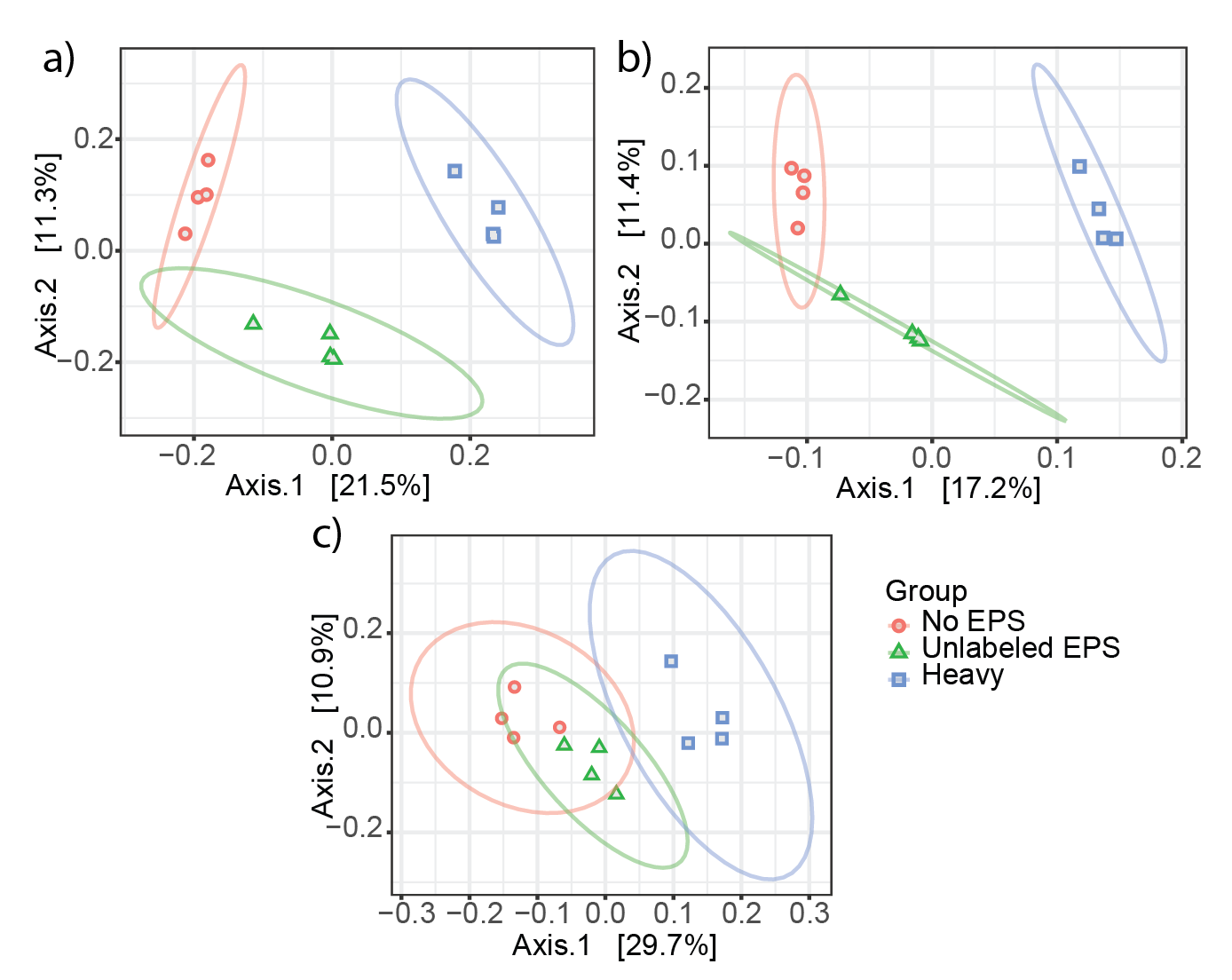

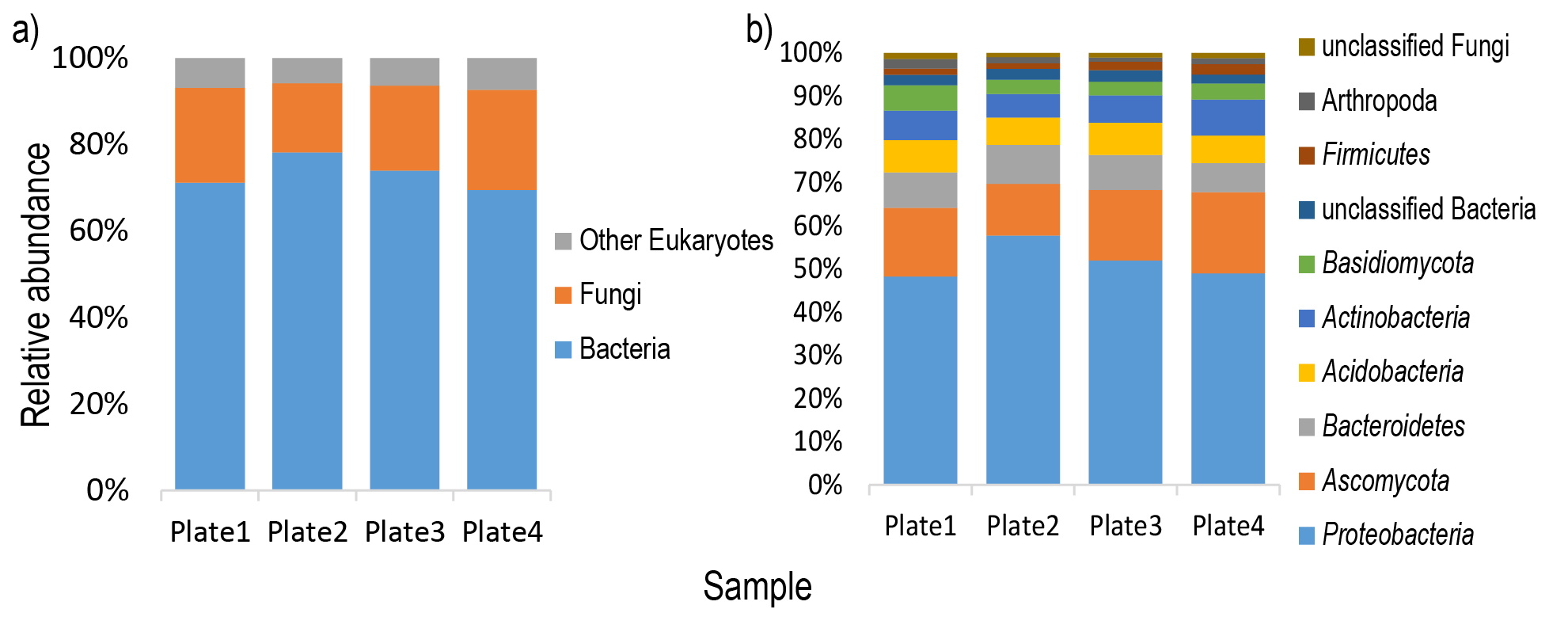


**Supplementary Figure S6:** Taxonomic composition and relative abundance of microbial groups at a) kingdom and b) phylum level in samples from the metagenome shotgun of cultivated microorganism based SSU rRNA gene taxonomic classification. Average from 2 replicates per plate of culture medium.

**Supplementary Figure S8:** Distribution of the 20 most abundant CAZyme families in a) SIP metagenome samples (relative abundance, average of 4 replicates); b) metagenome of cultivated microorganisms (relative abundance, average of 2 replicates); c) Metagenome-Assembled Genomes (MAGs) (number of genes), and most abundant glycosyl hydrolases (GH) in d) SIP metagenome samples (relative abundance, average of 4 replicates), e) metagenome of cultivated microorganisms (relative abundance, average of 2 replicates) and f) Metagenome-Assembled Genomes (MAGs) (number of genes).


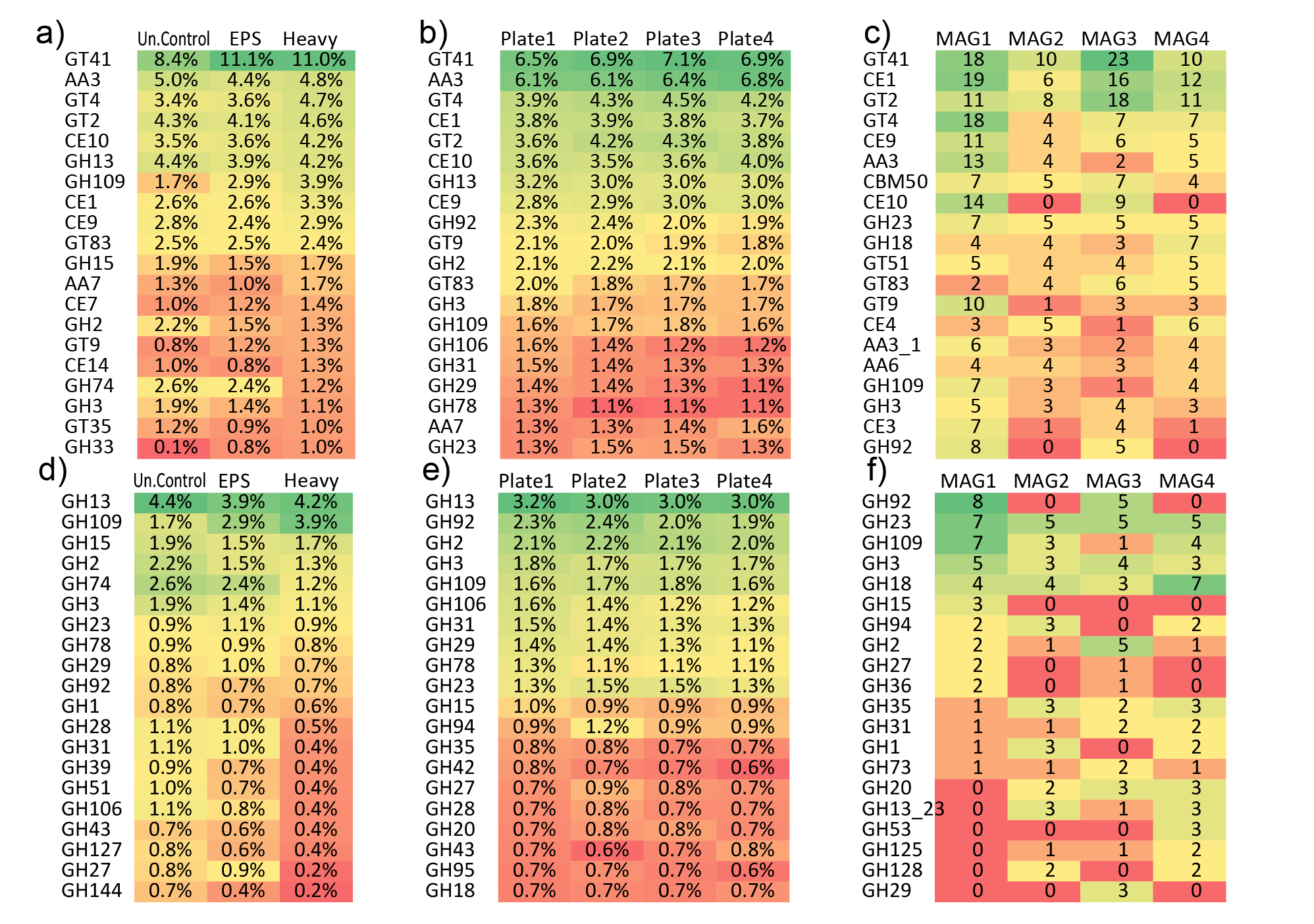

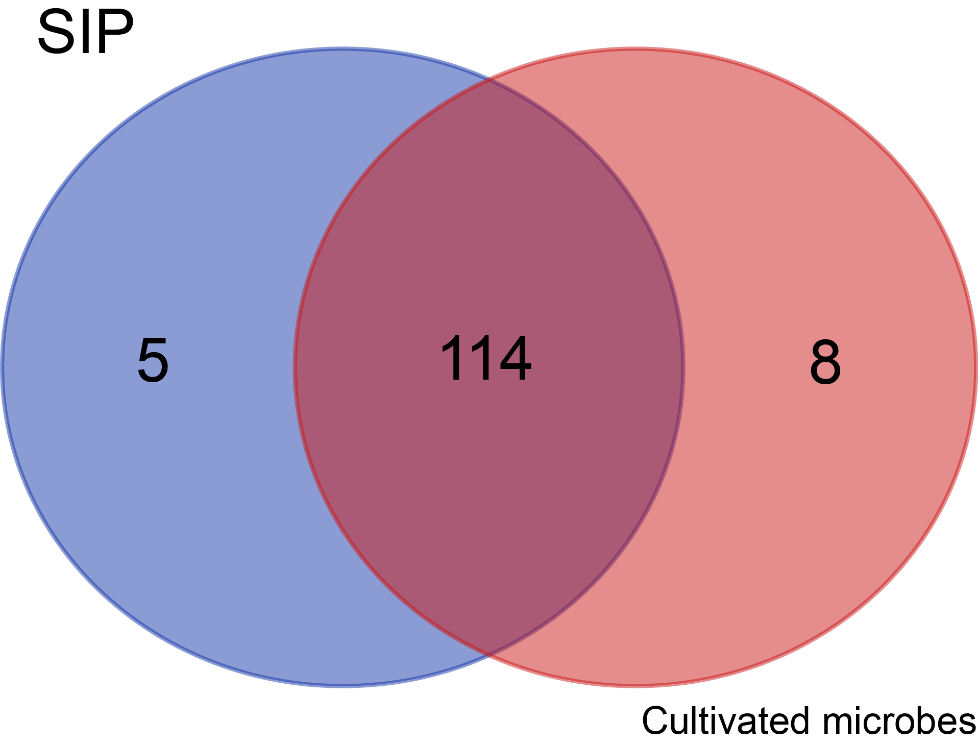


**Supplementary Figure S7:** Venn diagram depicting the number of common and unique glycoside hydrolase (GH) families observed in SIP metagenome and metagenome of cultivate microorganisms` datasets.


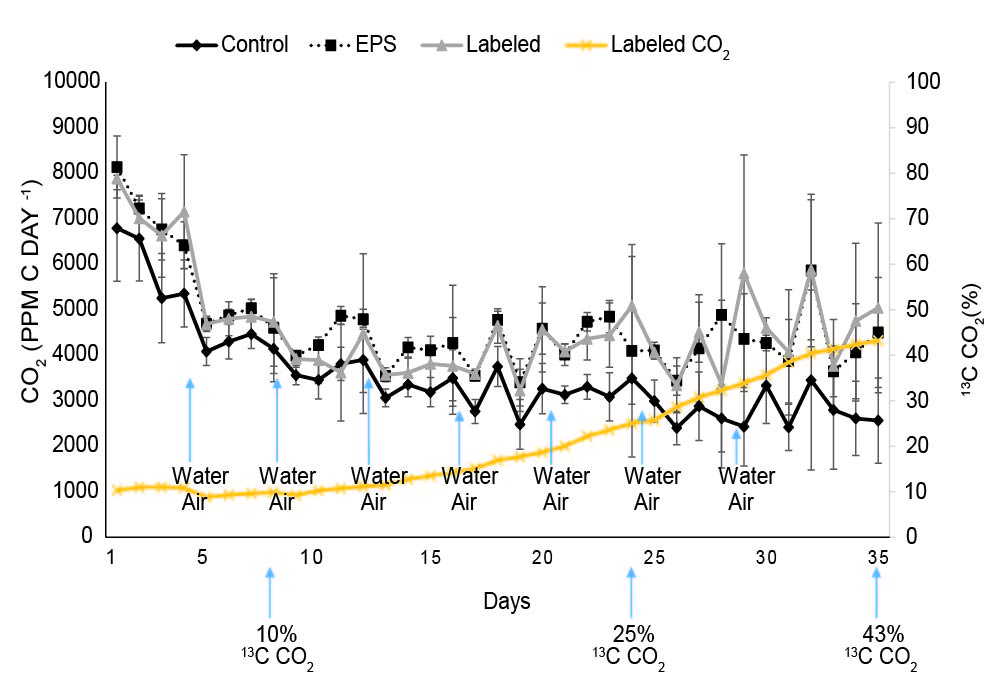


**Supplementary Figure S9:** CO_2_ emission. CO_2_ production during total incubation period. Control: control without EPS; EPS: control containing ^12^C-EPS; Labeled: incubation with ^13^C-EPS; Labeled CO_2_ percentage: ^13^CO_2_ emitted during ^13^C-EPS sample incubation; water: days when samples were hydrated; air: days when samples were aired.

| **Supplementary Table S1:** COG functions that significantly segregated across treatments selected by Boruta random forests algorithm based on 1000 permutations in the SIP metagenome treatment comparisons. | | | |
| --- | --- | --- | --- |
| **COG** | **Function** |  | **Category** |
| COG0043* | 3-polyprenyl-4-hydroxybenzoate decarboxylase | H | Coenzyme transport and metabolism |
| COG2985* | transport protein/Uncharacterized membrane protein YbjL | P | Inorganic ion transport and metabolism |
| COG3492* | Deoxycytidine triphosphate deaminase | S | Function unknown |
| COG3534 | Alpha-N-arabinofuranosidase (EC 3.2.1.55 | G | G Carbohydrate transport and metabolism |
| COG3547* | Transposase | L | Replication, recombination and repair |
| ENOG410XNWR | ABC transporter substrate-binding protein | E | Amino acid transport and metabolism |
| ENOG410XQPR* | Sulfite exporter TauE/SafE | S | Function unknown |
| ENOG410XQRG | Transcriptional regulator | K | Transcription |
| ENOG410XRW3* | secreted protein | S | Function unknown |
| ENOG410Y9A5* | Cna B-type protein | S | Function unknown |
| ENOG410YCZ9* | NA | S | Function unknown |
| ENOG410ZNCC* | NA | S | Function unknown |
| ENOG4111SDB* | NA | S | Function unknown |
| COG0665 | Glycine/D-amino acid oxidase | E | Amino acid transport and metabolism |
| COG0745 | OmpR -regulatoR | T | Signal transduction mechanisms |
| COG1309 | Transcriptional regulator | K | Transcription |
| COG2243 | Precorrin-2 methylase | H | Coenzyme transport and metabolism |
| ENOG410XPHN* | NA | S | Function unknown |
| ENOG410XQ2Q* | sialic acid-specific 9-O-acetylesterase | S | Function unknown |
| ENOG410XQ46 | Protein of unknown function (DUF1549) (*Planctomycetes*) | S | Function unknown |
| ENOG410XQWX* | NA | S | Function unknown |
| ENOG410XQXZ | NA | S | Function unknown |
| ENOG410XRSU | NA (*Acidobacteria*) | S | Function unknown |
| ENOG410XZSR | NA (*Acidobacteria*) | S | Function unknown |
| ENOG410Y0YH | reductase | I | Lipid transport and metabolism |
| ENOG410ZWC6 | kelch repeat-containing protein | S | Function unknown |
| ENOG4111HEH | (ABC) transporter | S | Function unknown |
| ENOG4111HR8 | Tetr family transcriptional regulator | K | Transcription |
| ENOG4111R18 | integral membrane transport protein | P | Inorganic ion transport and metabolism |
| ENOG4111TA9 | NA | S | Function unknown |
| ENOG41120VN | NA (*Planctomycetes*) | S | Function unknown |
| ENOG41126W5 | NA | S | Function unknown |
| *Higher abundance in ^12^C-EPS-amended control samples. Blue – more abundant in unamended control samples. Orange: more abundant in Labeled samples | | | |

| **Supplementary Table S2:** KEGG orthologs that significantly segregated across treatments selected by Boruta random forests algorithm based on 1000 permutations in the SIP metagenome treatment comparisons. | | | | |
| --- | --- | --- | --- | --- |
| **KEGG** | | **Annotation** | **KEGG Metabolic Pathway** | |
| K00467 | | Lactate 2-monooxygenase | Metabolic pathways/Pyruvate | |
| K01811* | | Alpha-D-xyloside xylohydrolase | - | |
| K01999* | | Branched-chain amino acid transport system substrate-binding protein | ABC transporters/quorum sensing | |
| K02083 | | Allantoate deiminase | Metabolic pathways/Microbial metabolism/Purines | |
| K02274* | | Cytochrome c oxidase subunit I | Metabolic pathways/Oxidative phosphorilation | |
| K03074 | | Preprotein translocase subunit secf | Protein export/Bacterial secretion | |
| K03307* | | Solute:Na+ symporter, SSS family | - | |
| K03832* | | Periplasmic protein tonb | - | |
| K07263* | | Zinc protease | - | |
| K07795 | | Putative tricarboxylic transport membrane protein | Two-component system | |
| K08964 | | Methylthioribulose-1-phosphate dehydratase | Metabolic pathways/Amino-acids | |
| K09758* | | Aspartate 4-decarboxylase | Metabolic pathways/Amino-acids | |
| K10826 | | Putative ABC transporter transmembrane protein | - | |
| K18900 | | Lysr family transcriptional regulator, regulator for bpeef and oprc | - | |
| K00200* | | Formylmethanofuran dehydrogenase subunit A | Metabolic pathways/microbial metabolism/carbon metabolism/methane metabolism | |
| K00249 | | Acyl-coa dehydrogenase | Antibiotics/fatty acids/secondary metabolites | |
| K00477 | | Phytanoyl-coa hydroxylase | Peroxysome | |
| K01087 | | Trehalose 6-phosphate phosphatase | Metabolic pathways/starch sucrose met | |
| K01133 | | Choline-sulfatase | - | |
| K01258 | | Tripeptide aminopeptidase | - | |
| K01515 | | ADP-ribose pyrophosphatase | Metabolic pathways/Purine metabolism | |
| K01992 | | ABC-2 type transport system permease protein | - | |
| K02551 | | 2-succinyl-5-enolpyruvyl-6-hydroxy-3-cyclohexene-1-carboxylate synthase | Metabolic pathways/Ubiquinone biosynthesis | |
| K02657 | | Twitching motility two-component system response regulator pilg | Two component system/Biofilm | |
| K03075 | | Preprotein translocase subunit secg | Bacterial secretion/Protein export | |
| K03167* | | DNA topoisomerase VI subunit B | - | |
| K03620 | | Ni/Fe-hydrogenase 1 B-type cytochrome subunit | Two componente system | |
| K05786 | | Chloramphenicol-sensitive protein rard | - | |
| K05970* | | Sialate O-acetylesterase | - | |
| K06606* | | 2-keto-myo-inositol isomerase | Metabolic pathways/microbial metabolism/inositol metabolism | |
| K08282 | | Non-specific serine/threonine protein kinase | - | |
| K08602* | | Oligoendopeptidase F | - | |
| K09684 | | Pucr family transcriptional regulator, purine catabolism regulatory protein | - | |
| K10301* | | F-box protein 21 | - | |
| K10714* | | Methylene-tetrahydromethanopterin dehydrogenase | Metabolic pathways/ Microbial metabolism/ Carbon metabolism/ Methane metabolism | |
| K11708* | | Manganese/zinc/iron transport system permease protein | ABC transporters | |
| K15342 | | CRISP-associated protein Cas1 | - | |
| K17329 | | N,N'-diacetylchitobiose transport system substrate-binding protein | ABC transporters | |
| K19169* | | DNA sulfur modification protein dndb | - | |
| K19542 | | MFS transporter, DHA2 family, tetracycline/oxytetracycline resistance protein | - | |
| *Higher abundance in EPS amended samples. Blue – more abundant in unamended control samples. Orange: more abundant in Labeled samples | | | | |
| **Supplementary Table S3:** CAZyme families that significantly segregated across treatments selected by Boruta random forests algorithm based on 1000 permutations in the SIP metagenome treatment comparisons. | | | |  |
| **CAZY family** | **Function** | | |  |
| AA1* | Multicopper oxidases | | |  |
| CE8* | Pectin methylesterase | | |  |
| GH18* | Chitinase | | |  |
| GH2 | β-galactosidase | | |  |
| GH27* | α-galactosidase | | |  |
| GH28* | Pectin polygalacturonase | | |  |
| GH3 | β-glucosidase/xylosidase | | |  |
| GH31* | α-glucosidases | | |  |
| GH36 | α-galactosidase/N-acetylgalactosaminidase | | |  |
| GH42* | β-galactosidases | | |  |
| GH51 | Hemicellulases | | |  |
| GH79 | Proteoglycanases | | |  |
| GH115* | Xylan α-glucuronidase | | |  |
| CBM66 | Fructan-binding modules | | |  |
| CBM9* | Xylan-binding modules | | |  |
| CE6 | Acetyl xylan esterase | | |  |
| GH109 | α-N-acetylgalactosaminidase | | |  |
| GH117 | α-1,3-L-neoagarooligosaccharide hydrolase | | |  |
| GH17 | 1,3;1,4-β-D-glucan endohydrolases | | |  |
| GH32 | Invertases | | |  |
| GH33* | Sialidases | | |  |
| GH50 | β-agarase | | |  |
| GH71 | α-1,3-glucanase | | |  |
| GT12* | [N-acetylneuraminyl]-galactosylglucosylceramide N-acetylgalactosaminyltransferase | | |  |
| GT4 | Mannosyltransferases/N-acetylglucosaminyltransferases | | |  |
| GT87 | Polyprenol-P-Man: α-1,2-mannosyltransferase | | |  |
| PL10* | Pectate lyase | | |  |
| AA-auxiliary activity; CE-carbohydrate esterase; GH-glycoside hydrolase; GT-glycoside transferase; PL-polysaccharide lyases. *Higher abundance in EPS-amendedsamples. Blue – more abundant in unamended control samples. Orange: more abundant in Labeled samples | | | |  |

| **Supplementary Table S4:** Most abundant CAZyme families (above 1% abundance) and most abundant KEGG orthologs (above 0.2% abundance) in the shotgun metagenome of cultivated microorganisms. | | | | | | |
| --- | --- | --- | --- | --- | --- | --- |
| **Category** | **Ave abundance (%)** | | | | | **Associated functions** |
| **CAZy family** |  | | | | |  |
| GT41 | 6.83% | | | UDP-GlcNAc: peptide β-N-acetylglucosaminyltransferase; UDP-Glc: peptide N-β-glucosyltransferase | | |
| AA3 | 6.35% | | | Glucose-methanol-choline (GMC) family of oxidoreductases, | | |
| GT4 | 4.24% | | | Mannosyltransferases/ N-acetylglucosaminyltransferases | | |
| CE1 | 3.78% | | | Large variety of substrates | | |
| GT2 | 3.96% | | | Cellulose synthase, chitin synthase, mannosyltransferase, glucosyltransferase, galactosyltransferase, rhamnosyltransferase | | |
| CE10 | 3.67% | | | Esterases acting on non-carbohydrate substrates | | |
| GH13 | 3.05% | | | α-glucosidase | | |
| CE9 | 2.93% | | | Deacetylation of N-acetylglucosamine-6-phosphate to glucosamine-6-phosphate | | |
| GH92 | 2.15% | | | α-mannosidase | | |
| GT9 | 1.97% | | | Lipopolysaccharide N-acetylglucosaminyltransferase/Heptosyltransferase | | |
| GH2 | 2.07% | | | β-galactosidase | | |
| GT83 | 1.80% | | | Undecaprenyl phospho-α-L-4-amino-4-deoxy-L-arabinose; dodecaprenyl phospho-β-galacturonic acid:lipopolysaccharide core α-galacturonosyl transferase | | |
| GH3 | 1.71% | | | β-glucosidases/galactosidases/xylosidases | | |
| GH109 | 1.69% | | | α-N-acetylgalactosaminidase | | |
| GH106 | 1.34% | | | α-L-rhamnosidase/rhamnogalacturonan α-L-rhamnohydrolase | | |
| GH31 | 1.40% | | | α-xylosidase/glucosidase/mannosidase | | |
| GH29 | 1.29% | | | α-fucosidase | | |
| GH78 | 1.16% | | | α-L-rhamnosidase/rhamnogalacturonan α-L-rhamnohydrolase | | |
| AA7 | 1.40% | | | Gluco/chito-oligosaccharide oxidase | | |
| GH23 | 1.39% | | | Peptidoglycan lyase | | |
| CBM50 | 1.02% | | | Bind to the N-acetylglucosamine residues in bacterial peptidoglycans and in chitin | | |
| GT51 | 1.08% | | | Murein polymerase | | |
| CE4 | 1.08% | | | De-acylation of polysaccharides | | |
| **KEGG ortholog** | | |  | |  | |
| K03296 | | | 0,50% | Hydrophobic/amphiphilic exporter-1 (mainly G- bacteria), HAE1 family | | |
| K02014 | | | 0,50% | Iron complex outermembrane recepter protein | | |
| K02004 | | | 0,46% | Putative ABC transport system permease protein | | |
| K00059 | | | 0,41% | 3-oxoacyl-[acyl-carrier protein] reductase | | |
| K03406 | | | 0,32% | Methyl-accepting chemotaxis protein | | |
| K01995 | | | 0,29% | Branched-chain amino acid transport system ATP-binding protein | | |
| K00128 | | | 0,28% | Aldehyde dehydrogenase (NAD+) | | |
| K18139 | | | 0,27% | Outer membrane protein, multidrug efflux system | | |
| K01996 | | | 0,27% | Branched-chain amino acid transport system ATP-binding protein | | |
| K18138 | | | 0,26% | Multidrug resistance, efflux pump MexAB-OprM | | |
| K08191 | | | 0,26% | MFS transporter, ACS family, hexuronate transporter | | |
| K02030 | | | 0,25% | Polar amino acid transport system substrate-binding protein | | |
| K02056 | | | 0,25% | Simple sugar transport system ATP-binding protein | | |
| K00249 | | | 0,25% | Acyl-CoA dehydrogenase | | |
| K03088 | | | 0,24% | RNA polymerase sigma-70 factor, ECF subfamily | | |
| K03762 | | | 0,24% | MFS transporter, MHS family, proline/betaine transporter | | |
| K01897 | | 0,24% | | Long-chain acyl-CoA synthetase | | |
| K02483 | | | 0,23% | Two-component system, OmpR family, response regulator | | |
| K01990 | | | 0,22% | ABC-2 type transport system ATP-binding protein | | |
| K08369 | | | 0,21% | MFS transporter, putative metabolite:H+ symporter | | |
| K03296 | | | 0,50% | Hydrophobic/amphiphilic exporter-1 (mainly G- bacteria), HAE1 family | | |
| AA-auxiliary activity; CE-carbohydrate esterase; GH-glycoside hydrolase; GT-glycoside transferase; PL-polysaccharide lyases | | | | | | |

| **Supplementary Table S5:** MAGs coverage in all samples | | | | |
| --- | --- | --- | --- | --- |
| **Sample** | **MAG1** | **MAG2** | **MAG3** | **MAG4** |
| OCP1 | 160.7 | 18.9 | 1805.1 | 13.2 |
| OCP2 | 126.8 | 29.4 | 1285.5 | 20.6 |
| OCP3 | 252.2 | 81.7 | 1826.3 | 51.1 |
| OCP4 | 119.9 | 58.3 | 1348.9 | 45.5 |
| OCP5 | 191.6 | 0.0 | 1715.5 | 0.0 |
| OCP6 | 127.2 | 48.0 | 1443.8 | 40.0 |
| OCP7 | 150.3 | 0.0 | 1376.4 | 0.0 |
| OCP8 | 178.4 | 90.3 | 1149.0 | 54.5 |

| **Supplementary Table S6:** Most abundant KEGG orthologs in MAGs and their associated functions. A selection of the top 10 most abundant KEGG orthologs in each genome is displayed. Annotation performed using eggNOG database. | | |
| --- | --- | --- |
| **KEGG** | **Associated function** | **Category** |
| K18139 | Outer membrane protein, multidrug efflux system | Transporters |
| K02029 | Polar amino acid transport system permease protein | Transporters |
| K03762 | MFS transporter, MHS family, proline/betaine transporter | Transporters |
| K08369 | MFS transporter, putative metabolite:H+ symporter | Transporters |
| K10441 | Ribose transport system ATP-binding protein | Transporters |
| K00059 | 3-oxoacyl-[acyl-carrier protein] reductase | Lipid metabolism |
| K03088 | rpoE | Transcription machinery |
| K02014 | Iron complex outermembrane recepter protein | Transporters |
| K03296 | Hydrophobic/amphiphilic exporter-1 (mainly G- bacteria), HAE1 family | Transporters |
| K01990 | ABC-2 type transport system ATP-binding protein | Transporters |
| K03286 | OmpA-OmpF porin, OOP family | Transporters |
| K02004 | Putative ABC transport system permease protein | Transporters |
| K16089 | Outer membrane receptor for ferrienterochelin and colicins | Transporters |
| K03406 | Methyl-accepting chemotaxis protein | Signal transduction |
| K00799 | Glutathione S-transferase | Metabolism of amino acids |
| K02660 | Twitching motility protein PilJ | Secretion system |
| K02030 | Polar amino acid transport system substrate-binding protein | Transporters |
| K02027 | Multiple sugar transport system substrate-binding protein | Transporters |
| K10440 | Ribose transport system permease protein | Transporters |
| K02483 | Two-component system, OmpR family, response regulator | Two-component system |
| K01996 | Branched-chain amino acid transport system ATP-binding protein | Transporters |
| K02056 | Simple sugar transport system ATP-binding protein | Transporters |
| K01995 | Branched-chain amino acid transport system ATP-binding protein | Transporters |

| **Supplementary Table S7:** Sugar transporters in MAG1 annotated with eggNOG database. | | |
| --- | --- | --- |
| **KEGG** | **Gene** | **Type of transporter** |
| K10111 | malK | Multiple sugar transport |
| K10112 | msmX | Multiple sugar transport |
| K10227 | smoE | Sorbitol/mannitol transport system |
| K10228 | smoF | Sorbitol/mannitol transport system |
| K10229 | smoG | Sorbitol/mannitol transport system |
| K10439 | rbsB | Ribose transport system |
| K10440 | rbsC | Ribose transport system |
| K10441 | rbsA | Ribose transport system |
| K10537 | araF | L-arabinose transport system |
| K10538 | araH | L-arabinose transport system |
| K10539 | araG | L-arabinose transport system |
| K10543 | xylF | D-xylose transport system |
| K10544 | xylH | D-xylose transport system |
| K10545 | xylG | D-xylose transport system |
| K10552 | frcB | Fructose transport system |
| K10553 | frcC | Fructose transport system |
| K10554 | frcA | Fructose transport system |
| K10559 | rhaS | Rhamnose transport system |
| K10560 | rhaP | Rhamnose transport system |
| K10561 | rhaQ | Rhamnose transport system |
| K10562 | rhaT | Rhamnose transport system |
| K17315 | gtsA | Glucose/mannose transport system |
| K17316 | gtsB | Glucose/mannose transport system |
| K17317 | gtsC | Glucose/mannose transport system |

| **Supplementary Table S8:** Sugar transporters in MAG2 annotated with eggNOG database. | | |
| --- | --- | --- |
| **KEGG** | **Gene** | **Type of transporter** |
| K06726 | rbsD | D-ribose pyranase |
| K10108 | malE | Maltose/maltodextrin transport system |
| K10109 | malF | Maltose/maltodextrin transport system |
| K10110 | malG | Maltose/maltodextrin transport system |
| K10111 | malK | Multiple sugar transport |
| K10112 | msmX | Multiple sugar transport |
| K10117 | msmE | Raffinose/stachyose/melibiose transport system |
| K10118 | msmF | Raffinose/stachyose/melibiose transport system |
| K10119 | msmG | Raffinose/stachyose/melibiose transport system |
| K10191 | lacK | Lactose/L-arabinose transport system |
| K10193 | togM | Oligogalacturonide transport system |
| K10228 | smoF | Sorbitol/mannitol transport system |
| K10229 | smoG | Sorbitol/mannitol transport system |
| K10234 | aglG | Alpha-glucoside transport system |
| K10235 | aglK | Alpha-glucoside transport system |
| K10236 | thuE | Trehalose/maltose transport system |
| K10237 | thuF | Trehalose/maltose transport system |
| K10238 | thuG | Trehalose/maltose transport system |
| K10240 | cebE | Cellobiose transport system |
| K10241 | cebF | Cellobiose transport system |
| K10242 | cebG | Cellobiose transport system |
| K10439 | rbsB | Ribose transport system |
| K10440 | rbsC | Ribose transport system |
| K10441 | rbsA | Ribose transport system |
| K10537 | araF | L-arabinose transport system |
| K10538 | araH | L-arabinose transport system |
| K10539 | araG | L-arabinose transport system |
| K10540 | mglB | Methyl-galactoside transport system |
| K10541 | mglC | Methyl-galactoside transport system |
| K10542 | mglA | Methyl-galactoside transport system |
| K10543 | xylF | D-xylose transport system |
| K10544 | xylH | D-xylose transport system |
| K10545 | xylG | D-xylose transport system |
| K10548 | - | Putative multiple sugar transport |
| K10550 | alsC | D-allose transport system |
| K10552 | frcB | Fructose transport system |
| K10553 | frcC | Fructose transport system |
| K10554 | frcA | Fructose transport system |
| K10558 | lsrA | AI-2 transport system |
| K10559 | rhaS | Rhamnose transport system |
| K10560 | rhaP | Rhamnose transport system |
| K10561 | rhaQ | Rhamnose transport system |
| K10562 | rhaT | Rhamnose transport system |
| K15770 | cycB | Arabinogalactan oligomer / |
| K15771 | ganP | Arabinogalactan oligomer / |
| K15772 | ganQ | Arabinogalactan oligomer / |
| K17204 | eryE | Erythritol transport system |
| K17207 | xltA | Putative xylitol transport |
| K17208 | ibpA | Inositol transport system |
| K17209 | iatP | Inositol transport system |
| K17210 | iatA | Inositol transport system |
| K17213 | inositol | Inositol transport system substrate-binding |
| K17214 | inositol | Inositol transport system permease |
| K17215 | inositol | Inositol transport system ATP-binding |
| K17241 | aguE | Alpha-1 4-digalacturonate transport |
| K17242 | aguF | Alpha-1 4-digalacturonate transport |
| K17244 | chiE | Putative chitobiose transport |
| K17245 | chiF | Putative chitobiose transport |
| K17246 | chiG | Putative chitobiose transport |
| K17315 | gtsA | Glucose/mannose transport system |
| K17316 | gtsB | Glucose/mannose transport system |
| K17317 | gtsC | Glucose/mannose transport system |

| **Supplementary Table S9:** General type transporters in MAG3 annotated with eggNOG database. | |
| --- | --- |
| **KEGG** | **Type of transporter** |
| K06147 | ATP-binding cassette subfamily B bacterial |
| K02004 | Putative ABC transport system permease |
| K02003 | Putative ABC transport system ATP-binding |
| K01992 | ABC-2 type transport system permease |
| K01990 | ABC-2 type transport system ATP-binding |
| K03286 | OmpA-OmpF porin OOP family |
| K11085 | ATP-binding cassette subfamily B bacterial |
| K16013 | ATP-binding cassette subfamily C bacterial |

| **Supplementary Table S10:** Sugar transporters in MAG4 annotated with eggNOG database. | | |
| --- | --- | --- |
| **KEGG** | **Gene** | **Type of transporter** |
| K06726 | rbsD | D-ribose pyranase |
| K10108 | malE | Maltose/maltodextrin transport system substrate-binding protein |
| K10109 | malF | Maltose/maltodextrin transport system permease protein |
| K10110 | malG | Maltose/maltodextrin transport system permease protein |
| K10111 | malK | Multiple sugar transport system ATP-binding |
| K10112 | msmX | Multiple sugar transport system ATP-binding |
| K10117 | msmE | Raffinose/stachyose/melibiose transport system substrate-binding protein |
| K10118 | msmF | Raffinose/stachyose/melibiose transport system permease protein |
| K10119 | msmG | Raffinose/stachyose/melibiose transport system permease protein |
| K10191 | lacK | Lactose/L-arabinose transport system ATP-binding protein |
| K10193 | togM | Oligogalacturonide transport system permease protein |
| K10227 | smoE | Sorbitol/mannitol transport system substrate-binding protein |
| K10228 | smoF | Sorbitol/mannitol transport system permease protein |
| K10229 | smoG | Sorbitol/mannitol transport system permease protein |
| K10234 | aglG | Alpha-glucoside transport system permease protein |
| K10235 | aglK | Alpha-glucoside transport system ATP-binding protein |
| K10236 | thuE | Trehalose/maltose transport system substrate-binding protein |
| K10237 | thuF | Trehalose/maltose transport system permease protein |
| K10238 | thuG | Trehalose/maltose transport system permease protein |
| K10240 | cebE | Cellobiose transport system substrate-binding protein |
| K10241 | cebF | Cellobiose transport system permease protein |
| K10242 | cebG | Cellobiose transport system permease protein |
| K10439 | rbsB | Ribose transport system substrate-binding protein |
| K10440 | rbsC | Ribose transport system permease protein |
| K10441 | rbsA | Ribose transport system ATP-binding protein |
| K10537 | araF | L-arabinose transport system substrate-binding protein |
| K10538 | araH | L-arabinose transport system permease protein |
| K10539 | araG | L-arabinose transport system ATP-binding protein |
| K10540 | mglB | Methyl-galactoside transport system substrate-binding protein |
| K10541 | mglC | Methyl-galactoside transport system permease protein |
| K10542 | mglA | Methyl-galactoside transport system ATP-binding protein |
| K10543 | xylF | D-xylose transport system substrate-binding protein |
| K10544 | xylH | D-xylose transport system permease protein |
| K10545 | xylG | D-xylose transport system ATP-binding protein |
| K10548 | - | Putative multiple sugar transport system |
| K10550 | alsC | D-allose transport system permease protein |
| K10552 | frcB | Fructose transport system substrate-binding protein |
| K10553 | frcC | Fructose transport system permease protein |
| K10554 | frcA | Fructose transport system ATP-binding protein |
| K10559 | rhaS | Rhamnose transport system substrate-binding protein |
| K10560 | rhaP | Rhamnose transport system permease protein |
| K10561 | rhaQ | Rhamnose transport system permease protein |
| K10562 | rhaT | Rhamnose transport system ATP-binding protein |
| K15770 | cycB | Arabinogalactan oligomer / maltooligosaccharide transport |
| K15771 | ganP | Arabinogalactan oligomer / maltooligosaccharide transport |
| K15772 | ganQ | Arabinogalactan oligomer / maltooligosaccharide transport |
| K17208 | ibpA | Inositol transport system substrate-binding protein |
| K17213 | inositol | Inositol transport system substrate-binding protein |
| K17214 | inositol | Inositol transport system permease protein |
| K17215 | inositol | Inositol transport system ATP-binding protein |
| K17241 | aguE | Alpha-1 4-digalacturonate transport system substrate-binding |
| K17242 | aguF | Alpha-1 4-digalacturonate transport system permease |
| K17244 | chiE | Putative chitobiose transport system substrate-binding |
| K17245 | chiF | Putative chitobiose transport system permease |
| K17246 | chiG | Putative chitobiose transport system permease |
| K17313 | treU | Trehalose transport system permease protein |
| K17315 | gtsA | Glucose/mannose transport system substrate-binding protein |
| K17316 | gtsB | Glucose/mannose transport system permease protein |
| K17317 | gtsC | Glucose/mannose transport system permease protein |
| K17324 | glpS | Glycerol transport system ATP-binding protein |
| K17325 | glpT | Glycerol transport system ATP-binding protein |

| **Supplementary Table S11:** Families of CAZymes observed in the MAGs, number of ORFs in each genome and associated enzymatic functions. | | | | | |
| --- | --- | --- | --- | --- | --- |
| **dbcan** | **MAG1** | **MAG2** | **MAG3** | **MAG4** |  |
| GH1 | 1 | 3 | 0 | 2 | β-glucosidases and β-galactosidases: |
| GH10 | 0 | 1 | 0 | 1 | Endo-beta-1,3-xylanase, endo-beta-1,4-xylanases |
| GH102 | 1 | 1 | 0 | 1 | Lytic transglycosidease |
| GH103 | 1 | 1 | 1 | 1 | Lytic transglycosideases |
| GH104 | 0 | 0 | 1 | 0 | Lytic transglyosylases |
| GH105 | 0 | 0 | 1 | 0 | Rhamnogalacturonidases |
| GH109 | 7 | 3 | 1 | 4 | α-N-acetylgalactosaminidase |
| GH12 | 1 | 0 | 0 | 0 | Endo-β-1,4-glucanase, endo-β-1,3-1,4-glucanase |
| GH123 | 0 | 0 | 1 | 0 | N-acetyl-β-galactosaminidases |
| GH125 | 0 | 1 | 1 | 2 | α-mannosidases |
| GH127 | 0 | 0 | 0 | 1 | Arabinofuranosidase |
| GH128 | 0 | 2 | 0 | 2 | β-1,3-glucanases |
| GH13_10 | 1 | 0 | 0 | 0 | α-hydrolases, transglycosidases and isomerases |
| GH13_11 | 1 | 1 | 1 | 1 | α-hydrolases, transglycosidases and isomerases |
| GH13_15 | 0 | 1 | 0 | 1 | α-hydrolases, transglycosidases and isomerases |
| GH13_16 | 1 | 0 | 0 | 0 | α-hydrolases, transglycosidases and isomerases |
| GH13_23 | 0 | 3 | 1 | 3 | α-hydrolases, transglycosidases and isomerases |
| GH13_26 | 1 | 0 | 0 | 0 | α-hydrolases, transglycosidases and isomerases |
| GH13_3 | 1 | 0 | 0 | 0 | α-hydrolases, transglycosidases and isomerases |
| GH13_36 | 0 | 1 | 1 | 1 | α-hydrolases, transglycosidases and isomerases |
| GH13_9 | 1 | 1 | 0 | 1 | α-hydrolases, transglycosidases and isomerases |
| GH130 | 0 | 0 | 1 | 0 | β-mannoside phosporilases |
| GH133 | 1 | 0 | 0 | 0 | Amylo-α-1,6-glucosidase |
| GH135 | 1 | 0 | 0 | 0 | Galactosaminogalactan hydrolase |
| GH141 | 1 | 0 | 0 | 0 | α-L-fucosidase/xylanase |
| GH144 | 0 | 1 | 0 | 1 | Endo-β-1,2-glucanase |
| GH15 | 3 | 0 | 0 | 0 | Glucoamylase |
| GH16 | 1 | 0 | 0 | 0 | β-1,4 or β-1,3 glycosidic bonds in various glucans and Galactans |
| GH17 | 0 | 0 | 2 | 0 | 1,3-β-D-glucan endohydrolases  and 1,3;1,4-β-D-glucan Endohydrolases |
| GH18 | 4 | 4 | 3 | 7 | Chitinases (EC 3.2.1.14) and endo-β-N-acetylglucosaminidase |
| GH19 | 0 | 0 | 1 | 0 | Chitinases |
| GH2 | 2 | 1 | 5 | 1 | β-galactosidases, β-glucuronidases, β-mannosidases, exo-β-glucosaminidases |
| GH20 | 0 | 2 | 3 | 3 | Exo-acting β-N-acetylglucosaminidases, β-N-acetylgalactosamindase and β-6-SO3-N-Acetylglucosaminidases |
| GH23 | 7 | 5 | 5 | 5 | Lytic transglycosideases |
| GH24 | 0 | 0 | 2 | 0 | Lysozyme |
| GH27 | 2 | 0 | 1 | 0 | α-Galactosidase |
| GH28 | 1 | 1 | 0 | 2 | Polygalacturonases. |
| GH29 | 0 | 0 | 3 | 0 | α-fucosidases |
| GH3 | 5 | 3 | 4 | 3 | Exo-acting β-D-glucosidases, α-L-arabinofuranosidases, β-D-xylopyranosidases |
| GH30 | 0 | 0 | 1 | 0 | β-glucosylceramidase, β-1,6-glucanase, and β-xylosidase |
| GH31 | 1 | 1 | 2 | 2 | α-glucosidases |
| GH33 | 0 | 1 | 1 | 1 | Sialidases and trans-sialidases |
| GH35 | 1 | 3 | 2 | 3 | β-galactosidases |
| GH36 | 2 | 0 | 1 | 0 | α-N-acetylgalactosaminidase |
| GH37 | 1 | 0 | 1 | 0 | Trehalase |
| GH42 | 1 | 0 | 1 | 1 | β-galactosidases, α-L-arabinosidase, β-D-fucosidase |
| GH49 | 1 | 0 | 0 | 0 | dextranase, isopullulanase |
| GH5 | 1 | 0 | 0 | 0 | Endo- and exoglucanases, endo- and exomannanases |
| GH5_13 | 1 | 0 | 0 | 0 | Endo- and exoglucanases, endo- and exomannanases |
| GH5_19 | 0 | 1 | 0 | 1 | Endo- and exoglucanases, endo- and exomannanases |
| GH5_43 | 1 | 0 | 0 | 0 | Endo- and exoglucanases, endo- and exomannanases |
| GH5_48 | 0 | 1 | 0 | 1 | Endo- and exoglucanases, endo- and exomannanases |
| GH50 | 0 | 0 | 1 | 0 | β-agarase |
| GH51 | 0 | 0 | 0 | 1 | L-arabinfuranosidases |
| GH53 | 0 | 0 | 0 | 3 | β-1,4-galactanase |
| GH54 | 2 | 0 | 0 | 0 | α-L-arabinofuranosidase and β-xylosidase |
| GH55 | 1 | 1 | 1 | 1 | β-1,3-glucanases, including both exo- and endo |
| GH63 | 1 | 1 | 1 | 1 | Exo-acting α-glucosidases |
| GH64 | 1 | 1 | 0 | 2 | β-1,3-glucanase |
| GH65 | 0 | 0 | 2 | 0 | Maltose phosphorylase, trehalose phosphorylase, kojibiose phosphorylase |
| GH73 | 1 | 1 | 2 | 1 | β-N-acetylglucosaminidases. |
| GH76 | 0 | 1 | 1 | 0 | Endo-acting α-mannanases |
| GH77 | 1 | 0 | 0 | 0 | 4-α-glucanotransferase |
| GH78 | 1 | 0 | 1 | 0 | α-L-rhamnosidases |
| GH79 | 1 | 0 | 1 | 0 | β-glucuronidase , β-4-O-methyl-glucuronidase , baicalin β-glucuronidase , heparanase and hyaluronidase |
| GH87 | 1 | 0 | 0 | 0 | α-1,3-glucanase |
| GH9 | 0 | 0 | 1 | 1 | Cellulases |
| GH92 | 8 | 0 | 5 | 0 | Exo-acting α-mannosidases, |
| GH94 | 2 | 3 | 0 | 2 | phosphorylase, cellodextrin phosphorylase, chitobiose phosphorylase |
| GH95 | 1 | 0 | 0 | 0 | 1,2-α-L-fucosidases, 1,2-α-L-galactosidases |

| Supplementary Table S12: Coordinates of the sampling sites | | |
| --- | --- | --- |
| Site number | **Latitude** | **Longitude** |
| 1 | 51°59'14.5“N | 5°47'32.7"E |
| 2 | 51°59'15.9"N | 5°47'29.5"E |
| 3 | 51°59'15.7"N | 5°47'27.7"E |
| 4 | 51°59'14.8"N | 5°47'23.2"E |

| **Supplementary Table S13:** Physicochemical properties of topsoil-litter samples. | | | | | |
| --- | --- | --- | --- | --- | --- |
| **Component** | **Unit** | **Average(Sd)** | **Component** | **Unit** | **Average(Sd)** |
| **total N** | **mg N/kg** | 16535±3217 | **CEC** | **%** | 81±0.0 |
| **C/N ratio** |  | 20±6 | **CEC** | **mmol+/kg** | 214±42 |
| **Available N** | **kg N/ha** | 252±187 | **B** | **μg B/kg** | 488±4.2 |
| **pH** |  | 3.05±0.1 | **Cu** | **μg Cu/kg** | 48±9.9 |
| **OM** | **%** | 55.8±3.5 | **Fe** | **μg Fe/kg** | 3070±466.7 |
| **Na** | **mg Na/kg** | 34.5±4.9 | **Mn** | **μg Mn/kg** | 101920±9362.1 |
| **P** | **mg P/kg** | 42.65±6.4 | **Zn** | **μg Zn/kg** | 8860±693.0 |
| **K** | **mg K/k** | 218±11.3 | **Clay** | **%** | 5.5±0.7 |
| **Ca** | **kg Ca/ha** | 13±0 | **Silt** | **%** | 10±14.1 |
| **Mg** | **mg Mg/kg** | 175±7.1 | **Sand** | **%** | 27.5±12.0 |

Sd: Standard deviation
